# Ontogeny and organ‐specific steroidal glycoside diversity is associated with differential expression of steroidal glycoside pathway genes in two *Solanum dulcamara* leaf chemotypes

**DOI:** 10.1111/plb.13704

**Published:** 2024-08-16

**Authors:** R. A. Anaia, I. Chiocchio, R. Sontowski, B. Swinkels, F. Vergara, N. M. van Dam

**Affiliations:** ^1^ Molecular Interaction Ecology, German Centre for Integrative Biodiversity Research (iDiv) Halle‐Jena‐Leipzig Leipzig Germany; ^2^ Institute of Biodiversity, Friedrich Schiller University Jena Germany; ^3^ Plant and Animal Biology Radboud Institute for Biological and Environmental Sciences, Radboud University Nijmegen the Netherlands; ^4^ Department of Pharmacy and Biotechnology University of Bologna Bologna Italy; ^5^ Leibniz Institute for Vegetable and Ornamental Crops (IGZ) Großbeeren Germany

**Keywords:** glycoalkaloids, LC–MS, mass‐difference networking, phytochemical diversity, qPCR, Saponins

## Abstract

- Solanaceous plants, such as *Solanum dulcamara*, produce steroidal glycosides (SGs). Leaf SG profiles vary among *S. dulcamara* individuals, leading to distinct phytochemical phenotypes (‘chemotypes’) and intraspecific phytochemical diversity (‘chemodiversity’). However, if and how SG chemodiversity varies among organs and across ontogeny, and how this relates to SG metabolism gene expression is unknown.
- Among organs and across ontogeny, *S. dulcamara* plants with saturated (*S*) and unsaturated (*U*) SG leaf chemotypes were selected and clonally propagated. Roots, stems and leaves were harvested from vegetative and flowering plants. Extracts were analysed using untargeted LC–MS. Expression of candidate genes in SG metabolism (*SdGAME9*, *SdGAME4*, *SdGAME25*, *SdS5αR2* and *SdDPS*) was analysed using RT‐qPCRs.
- Our analyses showed that SG chemodiversity varies among organs and across ontogeny in *S. dulcamara*; SG richness (*D*_*mg*_) was higher in flowering than vegetative plants. In vegetative plants, *D*_*mg*_ was higher for leaves than for roots.
- Lack of *SdGAME25* expression in *U*‐chemotype leaves, while readily expressed in roots and stems, suggests a pivotal role for *SdGAME25* in differentiation of leaf chemotypes in vegetative and flowering plants. By acting as an ontogeny‐dependent chemotypic switch, differential regulation of *SdGAME25* enables adaptive allocation of SGs, thereby increasing SG chemodiversity in leaves. This indicates that differential expression and/or regulation of glycoalkaloid metabolism genes, rather than their presence or absence, explains observed chemotypic variation in SG chemodiversity among organs and across ontogeny.

## Graphical abstract

Steroidal glycoside diversity among organs across ontogeny in relation to candidate gene expression in two *Solanum dulcamara* chemotypes.

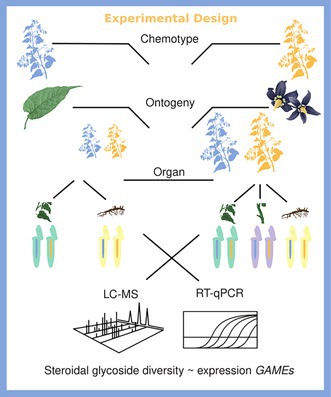

## INTRODUCTION

Plants chemically defend themselves by employing plant‐specialized metabolites (PSMs). Specifically, the *Solanum* produce PSMs called steroidal glycosides (SGs), which serve as chemical defence against herbivores (Calf *et al*. 2018) and pathogens (Sonawane *et al*. 2018). SGs consist of a steroidal aglycone (SA) that is conjugated to a glycoside moiety. This class includes steroidal saponin glycosides (SSGs) and their nitrogen‐containing analogues, steroidal glycoalkaloids (SGAs). As a class, SGs are structurally highly diverse, among others, because of variations in saturation of the steroidal aglycone and types and numbers of sugar molecules in the glycoside chain (Zhao *et al*. 2021). *Solanum* species, and individuals within a species, may have distinct SG profiles. In *S. dulcamara*, structural variation in SGs is based on variations in the aglycone, and their decoration, which give rise to intraspecific ‘phytochemical diversity’ (hereafter ‘chemodiversity’). These structural variations are introduced by chemical modifications, e.g. hydroxylation, acetylation and glycosylation, of the aglycone. These modifications may result from spontaneous or enzyme‐catalysed reactions (Wang *et al*. 2019). Furthermore, the steroidal aglycones may vary in number of double bonds and rings. This can be expressed as the ring‐double bond equivalent (RDBE), which is equal to the sum of the number of rings and double bonds in a molecular system (Heinig & Aharoni 2014). For instance, spirostanes consist of six rings (A – F), while furostanes have five‐membered ring system (A – E) in which the F‐ring is opened into an alkyl chain.

Over the last decade, great advances have been made in identification and characterization of genes involved in biosynthesis of SGs, including SSGs (Cheng *et al*. 2023) and SGAs (Itkin *et al*. 2013; Cárdenas *et al*. 2015; Sonawane *et al*. 2020), as well as in that of their precursor, cholesterol (Itkin *et al*. 2013; Sonawane *et al*. 2017). The majority of genes involved in SGA production are clustered on two chromosomes, which are syntenic across *S. lycopersicum* and *S. tuberosum* (Itkin *et al*. 2013), and thus likely wild relatives. Genes involved in production of SGA and SGG are commonly referred to as *GLYCOALKALOID METABOLISM* (*GAME*) genes. In *Solanum* spp., SGA biosynthesis is regulated by a transcription factor called Jasmonate‐Responsive Ethylene Response Factor 4 (JRE4) or GAME9 (Cárdenas *et al*. 2016; Nakayasu *et al*. 2018). A key enzyme in SGA biosynthesis is GAME4, which catalyses the first dedicated step in SGA production (Itkin *et al*. 2013; Paudel *et al*. 2017). In *S. lycopersicum*, β‐Hydroxysteroid Dehydrogenase/3‐Ketosteroid Reductase (3βHSD1) or GAME25, and STEROID 5α‐REDUCTASE2 (S5αR2) are both involved in reduction of the double bond between C5 and C6 in the B‐ring of dehydrotomatidine to produce tomatidine (Akiyama *et al*. 2019; Lee *et al*. 2019; Sonawane *et al*. 2018). After glycosylation of steroidal aglycones, spirostanes, such as (dehydro)tomatine, may potentially be further transformed into solanidanes. Spirostanes and solanidanes are six‐ringed steroids with distinct fusion patterns between their E and F rings. Spirostanes are fused by a single quaternary carbon atom (spiro carbon), whereas solanidanes are fused by a single covalent bond between a tertiary carbon and nitrogen atom, in an ortho fusion arrangement (Moss 1998). A 2‐oxoglutarate‐dependent dioxygenase, DIOXYGENASE FOR POTATO SOLANIDANE SYNTHESIS (DPS), catalyses C‐16α hydroxylation of spirostanes, which is considered the first‐dedicated step towards solanidane‐type SGAs in *S. tuberosum* (Akiyama *et al*. 2021).

Consequently, the observed SG chemodiversity in *S. dulcamara* may be related to the absence or presence of particular genes involved in SG biosynthetic pathways or to differential expression patterns (Calf *et al*. 2019). Interestingly, differences in the relative presence of SGAs with saturated (*S*) or unsaturated (*U*) steroidal aglycones have been related to differences in gastropod preference in preference assays with leaf discs (Calf *et al*. 2018). In both preference assays and a common garden experiment, plants that predominantly produce *S*‐type SGAs were more preferred by generalist slugs (*Deroceras reticulatum*) compared to *U*‐type SGAs containing accessions (Calf *et al*. 2018, 2019). On the other hand, specialist flea beetles were more abundant on plants with *U*‐type SGA profiles, and avoided plants rich in SSGs rather than SGAs (Calf *et al*. 2019). It was postulated that SG leaf chemotypes in *S. dulcamara* may be heritable (Schreiber & Rönsch 1965; Willuhn 1966; Calf 2019). This suggests that SG chemodiversity in *S. dulcamara* may be driven by differential selection pressures exerted by different herbivore communities (Wetzel & Whitehead 2020; Petrén *et al*. 2023a, 2023b; Thon *et al*. 2024).

Although SG chemodiversity in *S. dulcamara* leaves (Calf *et al*. 2018) and roots (Chiocchio *et al*. 2023) is well described, little is known about ontogenetic, organ‐ and chemotype‐specific SG variation in *S. dulcamara*, especially in relation to expression of candidate genes in SG biosynthesis. It is known from other PSM and families, e.g. glucosinolates in Brassicaceae, that leaf and root profiles differ considerably within individual plants (van Leur *et al*. 2006; Tsunoda *et al*. 2017). onsequently, leaf glucosinolate profiles are more distinct than these of roots in two different *Barbarea vulgaris* leaf chemotypes (van Leur *et al*. 2008). Recently, we profiled SG chemodiversity in embryonic and adventitious roots of *S. dulcamara*, using liquid chromatography coupled to mass spectrometry (LC–MS). We found that both root types have distinct SG profiles (Chiocchio *et al*. 2023). This suggests that there may be additional levels of intraspecific and intra‐individual chemical diversity than in leaves. Based on these root analyses, we proposed a SG classification system for mass spectra, based on structural differences among steroidal aglycones (Chiocchio *et al*. 2023). Such classification systems allow studying SGs in terms of chemodiversity, since we can apply (species) diversity indices, such as Margalef's richness (Margalef 1958) and Pielou's evenness (Pielou 1966), to PSMs as previously suggested (Hilker 2014; Marion *et al*. 2015; Kessler & Kalske 2018; Wetzel & Whitehead 2020; Petrén *et al*. 2023a, 2023b; Thon *et al*. 2024). For SGs, chemodiversity can be measured in terms of chemical richness and evenness, by considering the existence of distinct ‘steroidal aglycone species’ and the number of associated glycosides per unique aglycone species. Quantifying chemical richness among organs and individual leaf chemotypes in conjunction with expression of relevant genes, enhances our understanding of how chemodiversity is regulated within plants as well as in ontogenetic development. Moreover, it provides new hypotheses on potential selection processes that have shaped evolution of different levels of chemodiversity.

Here, we analysed whether SG profiles are organ‐specific in vegetative and flowering *S. dulcamara* full‐sibs of two contrasting leaf chemotypes. We asked whether the SGA leaf chemotype (hereafter ‘chemotype’) is constant over plant organs and ontogeny. Furthermore, we investigated whether the previously defined SGA chemotypes are characterized by a broader SG diversity using chemical profiling across ontogenetic stages and organs within a plant. Thereafter, we asked whether the detected organ, ontogeny and chemotype‐specific variation in SG chemodiversity is related to the differential expression of candidate genes in SG metabolism. To do so, we prepared an F1 population by crossing two Dutch *S. dulcamara* accessions: ‘Zandvoort Dry’ (ZD04) and ‘Texel Wet’ (TW12) described by Calf *et al*. (2018). Accession ZD04 produces unsaturated SGAs in leaves, while accession TW12 predominantly produces saturated SGAs. The F1 progeny (TW12 × ZD04) was chemotyped by LC–MS, after which siblings with *S*‐ or *U*‐chemotypes were selected for further analyses. The selected plants were chemically profiled for SG chemodiversity in adventitious roots, stems and leaves of vegetative and flowering *S. dulcamara*. In the same tissues, we assessed expression of candidate genes in SG biosynthesis, including homologues of *GAME9* (Cárdenas *et al*. 2016), *GAME4* (Itkin *et al*. 2013; Paudel *et al*. 2017), *GAME25* (Lee *et al*. 2019; Sonawane *et al*. 2018). *S5αR2* (Akiyama *et al*. 2019) and *DPS* (Akiyama *et al*. 2021) using RT‐qPCR. Together, these analyses provide new insights into regulation of chemotypic, organ‐ and ontogeny‐specific SG chemodiversity in two main *S. dulcamara* chemotypes.

## MATERIAL AND METHODS

### Plant material, chemotyping and experimental design

Hybrid *S. dulcamara* (TW12 × ZD04) seeds were germinated as described in Chiocchio *et al*. (2023). Briefly, seeds were placed onto wet glass beads (1 mm Ø) in plastic boxes. Seeds were cold stratified in the dark at 4 °C for 2 weeks. Then, the seed‐containing plastic boxes were put in a climate chamber (L:D 16 h:8 h, 20 °C day/17 °C night, with light of 500 μmol·m^−2^·s^−1^) to induce germination (Chiocchio *et al*. 2023). Emerged seedlings with two similarly sized cotyledons were transplanted to trays (QuickPot™ 24R, Ø 7.5 × 7.0 cm; Groß Kreutz, Germany) filled with a 1:1 (v/v) autoclaved soil (Floradur B pot clay medium coarse; Floragard Vetriebs, Germany) and sand (0/2 washed; Rösl Rohstoffe, Germany) mixture. When seedlings had a second set of true leaves, leaf samples were taken for metabolite extraction and SG chemotyping, as described below. For chemotyping, extracted ion chromatograms (EICs) were produced for *m/z* 414.3 and *m/z* 416.3 and the resulting EICs were inspected. Plants were assigned to the saturated (*S*) SG chemotype when peak *m/z*‐fragment 416.3 was present in the chromatograms, while plants were assigned to the unsaturated (*U*) SG chemotype when *m/z* 414.3 was observed in the absence of *m/z* 416.3. Based on available plant material, three *S*‐chemo‐genotypes and four *U*‐chemo‐genotypes were selected for further experimentation.

To generate sufficient plant material for experimentation, multiple stem cuttings were taken from the chemotyped stock plants, as described by Calf *et al*. (2018). Stem cuttings were potted (11 × 11 × 12 cm) in 1 l pots containing a well‐watered 1:1 soil:sand mixture supplied with 4 g·l^−1^ Osmocote Pro 8‐9M (ICL Boulby, Cleveland, UK), and kept under greenhouse conditions (17–25 °C, RH: ±65%) with light supplemented to 280 μmol·m^−2^·s^−1^ with high‐pressure sodium lamps. Healthy, vigorously growing plants were selected for sampling 6 and 11 weeks after transplantation. At 6 weeks old, all plants were vegetative, with vegetative meristems and no inflorescences (vegetative stage), while 11‐week‐old plants were flowering, with open, pollen‐producing flowers (flowering). Leaves and adventitious roots (hereafter referred to as roots) were harvested from vegetative and flowering plants. In addition, stems of flowering plants were harvested. This was not possible for vegetative plants, as the remaining stems were used to generate new plants by clonal propagation, as described above. At harvest, plants were carefully removed from their pots and remaining soil removed under running tap water. Then roots were washed using deionized water and gently tapped dry with paper tissues. Simultaneously, five fully‐expanded leaves, counted from the first fully expanded (~2‐cm wide) leaf from the stem apex (Viswanathan & Thaler 2004), were harvested using sharp scissors. The leaves were stacked and midveins were removed with scissors. The stem segment on which the first five fully expanded leaves were present was cut into multiple pieces and sampled. Material for every harvested sample was divided and separately collected into two 15‐ml Falcon tubes, one for chemical and one for gene expression analyses, and immediately flash‐frozen in liquid nitrogen. Scissors were cleaned with 70% EtOH and tissue paper between harvests of different organ samples to avoid cross‐contamination.

### Sample processing and extraction of endogenous semi‐polar metabolites for metabolomic analysis

First, samples were freeze‐dried under vacuum to constant weight for 3 days in a freeze drier (FreeZone Plus 12; Labconco, Kansas City, MI, USA) at −80 °C. Thereafter, dried samples were ground using a ball mill (Mixer Mill MM 400; Retsch) containing two metal beads (5 mm Ø; 50 Hz, 3 × 10 s). Ground samples were stored in 2‐ml Safe‐Lock® tubes (Eppendorf, Hamburg, Germany) at room temperature. Aliquots of 20 ± 1 mg (leaves and stems) and 10 ± 1 mg (roots) were weighed into 2 ml round‐bottom Eppendorf tubes for metabolite extraction. Some root samples of vegetative plants weighed <10 mg; in those cases, extraction buffer volume was adjusted proportionally to mass of the sample. The samples were extracted using the protocol described in Chiocchio *et al*. (2023). Briefly, samples were extracted twice in 1 ml (leaves and stems) and 0.5 ml (roots) 3:1 methanol:acetate buffer (pH 4.8) in 2 ml reaction tubes containing metal beads by shaking in a ball mill (Mixer Mill MM 400; Retsch) at 50 Hz for 5 min. Thereafter, samples were centrifuged for 15 min at 15,000 *g* at 4 °C. Supernatants (~ 0.8 ml) of both extraction steps were combined and centrifuged for 10 min at 15,000 *g*. Dilutions of 1:10 (leaves, stems) and 1:5 (roots) were prepared by pipetting aliquots into amber 1 ml HPLC vials containing extraction buffer.

### Chemotyping and metabolomic profiling using UPLC‐qToF‐MS

Metabolomic profiling of semi‐polar metabolites was conducted as described by Chiocchio *et al*. (2023). Extracts were injected into a UPLC–MS (Dionex UltiMate 3000; Thermo Fisher Scientific, Waltham, USA) equipped with a C18 analytical column (Acclaim TM RSLC 120; 2.1 × 150 mm, 2.2 μm particle size, 120 Å pore size). The column was maintained at a constant temperature of 40°C. Mobile phases used consisted of water/formic acid (0.05% v/v, solvent A), and acetonitrile/formic acid (0.05% v/v, solvent B). The flow rate was 400 μl·min^−1^. The multi‐step gradient for solvent B was: 0–1 min 5%, 1–4 min 28%, 4–10 min 36%, 10–12 min 95%, 12–14 min 95%, 14–18 min 5%. The chromatograph was equipped with an autosampler that maintained samples at a constant temperature of 4°C and injected sample volumes of 1 μl (leaves and stems) or 2 μl (roots). The chromatograph was coupled with a maXis impact HD MS‐qToF (Bruker Daltonics, Bremen, Germany) operated in positive polarity. ESI source conditions were: end plate offset = 500 V, capillary = 4500 V, nebulizer = 2.5 bar, dry gas = 11 L·min^−1^, dry temperature = 220 °C. Transfer line conditions were: funnels 1 and 2 = RF 300 Vpp, isCD energy = 0 eV, hexapole = 60 Vpp, quadrupole ion energy = 5 eV, low mass = 50 *m/z*, collision cell energy = 10 eV, collision RF = 500 Vpp, transfer time = 60 μs, pre‐pulse storage = 5 μs. The mass spectrometer was operated in full scan (MS1) mode with a mass range of 50–1500 *m/z* and a spectral acquisition rate of 3 Hz. Masses were calibrated using sodium formate (10 mM) clusters, prepared by combining 250 ml isopropanol, 1 ml formic acid, and 5 ml 1 M sodium hydroxide. The mixture was adjusted to a final volume of 500 mL with water.

### Selection of candidate *GAME* genes and primer design

The *GAME9* transcribes a transcription factor that regulates *GAME* and upstream mevalonate pathway genes (Cárdenas *et al*. 2016) and its expression was used as an indicator of overall SGA biosynthetic activity. *GAME4* transcribes a cytochrome P450 that is active at the bifurcation step for biosynthesis of SGAs and SSGs (Paudel *et al*. 2017) and its expression was used as proxy for the influx of SA precursors into the SGA pathway. Expression of *GAME25* (Lee *et al*. 2019; Sonawane *et al*. 2018) and *S5αR2* (Akiyama *et al*. 2019) were used as proxies for conversion of unsaturated steroidal aglycones into saturated aglycones by GAME25 and S5αR2. Lastly, *DPS* expression was used as proxy for potential conversion of spirostanes into solanidane‐type SGAs (Akiyama *et al*. 2021). In addition, primers for reference genes, *SdEXP* (Expressed sequence) and *SdSAND* (a SAND family gene), were selected from their use in literature (Calf *et al*. 2019, 2020). For primer design, cDNA sequences of abovedescribed genes‐of‐interest (GOIs) functionally characterized in *S. tuberosum* and *S. lycopersicum* were queried against a *S. dulcamara* transcriptome (D'Agostino *et al*. 2013) using BLAST. All alignments with identity >40% were further examined by determining and translating their longest open reading frame (ORF) using the Expasy ‘Translate’ tool (Gasteiger *et al*. 2003). Then, amino acid sequences were aligned with the protein sequence of the GOI using CLUSTAL Omega (Sievers *et al*. 2011). This multiple sequence alignment was visually inspected to select the best homologue among the selected contigs. Selected *S. dulcamara* homologues of the GOI were fed into the NCBI Primer BLAST (Altschul *et al*. 1990) by uploading the relevant FASTA sequences and the *S. dulcamara* transcriptome (D'Agostino *et al*. 2013). The search parameters were left unchanged, except for PCR product size (75–200, Min–Max), Tm (58.0–60.0–62.0, Min–Opt–Max), primer GC content (45.0–65.0%, Min to Max) and Max Poly‐X (4). In addition, Primer3 was used to generate additional candidate primer pairs (Untergasser *et al*. 2012). The designed primer pairs were tested for specificity by evaluating the NCBI Primer BLAST results and using the BLAST tool of Solgenomics. Subsequently, the OligoAnalyzer tool (https://eu.idtdna.com/pages/tools/oligoanalyzer) of Integrated DNA Technologies (Coralville, IA, USA) was utilized to assess thermodynamic stability of any predicted secondary structures formed by the most selective primer pairs. The parameters of the analysis tool were adjusted to fine‐tune it for use on RNA sequences meant for PCR. A cut‐off of −9 kcal·mol^−1^ was used for the ΔG value, where any structure predicted with a ΔG below −9 kcal mol^−1^ resulted in rejection of the corresponding primer pair. The following gene specific primers were used for RT‐qPCRs of GOIs: *SdGAME9*‐F: GTGGTGTGTGAGGAAAACGC, *SdGAME9*‐R: CTCGGATCTTGTAAGCGGCT; *SdGAME4*‐F: ACGGGTTCTTCTGTAGCAGC, *SdGAME4*‐R: TCTCGGCGATTAACAGCTCC; *SdGAME25*‐F: TCTTGGCGTCCGATGAATCC, *SdGAME25*‐R: ACAGCACACCAACGAGAGAG; *SdS5αR2*‐F: GACCCGAATAAGACCAGCCC, *SdS5αR2*‐R: TACCCTCTTCGCCTCCACTT; *SdDPS*‐F: TGGTTTTAGAGAGTCTTGGGCT, *SdDPS*‐R: CCACCATCTGTGTGGCTACC; *SdSAND*‐F: TGCTTACACATGTCTTCCACTTGC, *SdSAND*‐R: AAACAGGACCCCTGAGTCAGTTAC and *SdEXP*‐F: CTAAGAACGCTGGACCTAATGACAAG, *SdEXP*‐R: AAAGTCGATTTAGCTTTCTCTGCATATTTC.

### RNA extraction and gene expression analysis using RT‐qPCRs

Fresh plant material was stored in 15 ml Falcon tubes at −80 °C until sample processing. Frozen plant tissues were ground to a fine powder under liquid nitrogen using a mortar and pestle. The ground samples were stored in 1.5 ml Eppendorf tubes at −80 °C. Total RNA was extracted from ground plant material according to a protocol adapted from Oñate‐Sánchez & Vicente‐Carbajosa (2008). Extracted RNA samples were treated with DNAase I (Thermo Fisher Scientific) according to the manufacturer's instructions. RNA integrity was visually inspected using gel electrophoresis. To check for RNA quality, absorbance ratios 260/230 and 260/280 nm were measured using a P330 NanoPhotometer® (IMPLEN, Munich, Germany) and quality checks were passed at absorbance ratios of in the ranges ~2–2.2 and ~1.8–2, respectively. Thereafter, 2 μg of DNA‐free RNA were transferred to a new 0.2 ml PCR tube containing 24 μl autoclaved ddH_2_O. Subsequently, 1 μl 50 μm Oligo dT 20 was added, after which the mixture was spun down. Thereafter, 4 μl of 5× RT buffer, 2 μl 10 mm dNTP Mix and 1 μl RevertAid H Minus Reverse Transcriptase (Thermo Fisher Scientific) were added, and the tube spun down again. Samples were incubated in a thermocycler (Techne, Stone, UK) for 60 min at 42 °C, 15 min at 50 °C and 15 min at 70 °C. Each sample was measured in triplicate following RT‐qPCR procedures on the CFX384 Real‐time system (Bio‐Rad, Munich, Germany), using 1 μl cDNA, 10 μl DreamTaq polymerase (DreamTaq Green PCR Master Mix 2×; Thermo Fisher Scientific), 0.5 μl 10 μm forward and reverse primers and 8 μl autoclaved ddH_2_O in a total volume of 20 μl per reaction. The qPCR conditions were: 10 s at 95 °C and 30 s at 60 °C for 40 cycles.

### Data processing and statistical analyses

All statistical analyses and data visualizations were performed and produced in R (version 4.3.1; R Core Team 2021), except when explicitly mentioned otherwise. Data visualizations were performed using R package *ggplot2* (Wickham 2016). In general, Linear Mixed Models (LMMs) were built using packages *lme4* and *glmmTMB* (Brooks *et al*. 2017). Model fit and residual diagnostics were checked using the *performance* (Bates *et al*. 2015) and *DHARMa* (Hartig 2022) packages.

A peak‐intensity table was produced by simultaneous pre‐processing all LC–MS data in MetaboScape 5 (Bruker Daltonics, Bremen, Germany). The resulting table was sum‐normalized, log‐transformed and mean‐centred, after which principal components analysis (PCA) was performed using MetaboAnalystR 4.0 (Pang *et al*. 2021).

A mass‐difference network (MDN) was inferred using the *MetNet* package (Naake & Fernie 2019). Briefly, commonly observed neutral losses (NLs) in SGs (Heinig & Aharoni 2014) including hydroxylation, glycosylation, acetylation and malonylation, were used to cluster nodes (which represent ions of specific *m/z*) associated with SGs. Then, a retention time‐corrected adjacency matrix based on structural information was built. Undirected network graphs were produced from the structural adjacency matrix and exported to .graphml format using R package *igraph* (Csardi & Nepusz 2006). Singleton nodes were removed from the MDN. Then, the MDN was visualized using Cytoscape version 3.8.2 (Shannon *et al*. 2003), and relative intensities in LC–MS were mapped onto its nodes. Mass spectra were manually exported from Bruker Data Analysis (v. 5.2 Bruker Daltonics) and wre plotted using SciDAVis version 2.4.0. Chemical structures of putative metabolites were drawn using ACD/ChemSketch version 2020.1.2 (Advanced Chemistry Development, Toronto, Ontario, Canada).

The MDN was manually inspected for *m/z* signals associated with steroidal aglycones, and glycosylated steroidal aglycones. Then, ion chromatograms (EICs) were manually extracted using Bruker Data Analysis (v.5.2; Bruker Daltonics) for 12 *m/z* values associated with steroidal aglycons (Fig. 2c; hereafter ‘SA species’): 412.32, 414.35, 416.35, 428.32, 430.33, 432.35, 415.32, 417.33, 433.33, 434.36, 446.33 and 448.34. Mass spectra associated with peaks in EICs were inspected for neutral losses indicative of glycosylation with pentoses, deoxyhexoses, or hexoses. Only peaks with a glycosylation signature were retained and counted. When multiple of the aforementioned SA species were found in the same mass spectrum, only the heaviest fragments were counted. The resulting matrix, containing the counted number of SGs per SA species for every chromatogram, was used to calculate diversity indices including Margalef's richness (Margalef 1958; $D_{\mathrm{mg}}$) and Pielou's evenness (Pielou 1966; J) which were defined as:

$$\text{Margale}f^{\prime }s\;\text{richness}=D_{\mathrm{mg}}=\frac{\left(S-1\right)}{\ln\left(N\right)}$$

where S is number of unique SA species, and N is total number of SG molecules detected in a chromatogram; and

$$\text{Pielo}u^{\prime }s\;\text{evenness}=J=\frac{H^{\prime }}{H_{\max}^{\prime }}=\frac{H^{\prime }}{\ln\left(S\right)}=-\frac{\sum_{i}^{S}p_{i}\ln\left(p_{i}\right)}{\ln\left(S\right)}$$

with $p_{i}=n_{i}/N$, where $n_{i}$ is the number of SG molecules belonging to the$\;i_{\mathrm{th}}$ SA species, N is the total number of SG molecules detected in a chromatogram, and S is number of unique SA species (i.e. J is equal to the ratio of the sample‐specific Shannon‐index ($H^{\prime }$; Shannon 1948) and its maximum value ($H_{\max}^{\prime }$).

The LMMs were built using SG richness and evenness as response variables. Fixed effects were modelled as the interaction between ‘chemotype’ and ‘organ, while ‘plant individual’ nested within ‘genotype’ was modelled as random effect. The Wald test was used to test the significance of predictors in models, with SG richness and evenness as responses. For post‐hoc testing, the estimated marginal means (EMM) method was applied to calculate EMMs for Margalef's richness ($D_{\mathrm{emm}}$) and Pielou's evenness ($J_{\mathrm{emm}}$) for treatment groups using the R package *emmeans* (Lenth 2024). TIC of SG‐associated features (TIC_SG_) was calculated by taking the sum of the intensities of ions in the MDN (excluding signals with *m/z* 329.32). Pearson correlation coefficient was used to investigate the relationship between TIC_SG_ and SG chemodiversity.

In order to partition the observed variation in SG counts for every experimental factor, two separate generalized linear models (GLM) of the Poisson family were built using the R package *gASCA* (Franceschi 2022). For the first GLM, organ‐type, chemotype and their interaction were specified as fixed effects. For the second GLM, sample‐type (defined as different organ–chemotype combinations), ontogeny and their interaction were specified as fixed effects. Models were subsequently used for anova‐simultaneous component analysis (ASCA). The decomposition was validated using a permutation‐based approach (n = 1000). Variables with higher *R* ^2^_pseudo_ in the specified models compared to their respective null‐model were selected for decomposition in the presented GLM‐ASCA models.

For RT‐qPCR data, generalized linear mixed models (GLMMs) of the Poisson family were built in a Bayesian framework using the R package *MCMC.qpcr* (Matz *et al*. 2013). For *SdDPS*, *SdGAME4*, *SdGAME9*, *SdGAME25*, and *SdS5αR2*, amplification efficiencies were calculated based on dilution series (1–1000×), and for *SdEXP* and *SdSAND* efficiencies of 2 were assumed and were used to inform the model. First, raw Cq values from RT‐qPCR were transformed into counts using the function *ct2counts()*. The MCMC chain in *mcmc.qpcr()* was set to 110,000 iterations, with a thinning interval of 100, and the initial 10000 iterations were discarded. Model convergence was inspected by checking the column ‘eff.samp’ in the model summary for values smaller than the difference between the total number of iterations and the number of discarded iterations, divided by the thinning interval (Matz *et al*. 2013).

To check for global effects, a naïve model containing all RT‐qPCR data was built. First, log_2_‐transformed data were extracted from the naïve model. Then, Manhattan distance indices were calculated from normalized data and were used as input for PERMANOVA and PCoA, using the functions *adonis2()* and *cmdscale()* from the R packages *vegan* and *stats*, respectively. Thereafter, separate soft‐normalization models were built per ontogenetic group using the *mcmc.qpcr()* function. Precisely, the interaction between the factors “target gene”, “chemotype”, and “organ” was specified as fixed effect, while “sample‐type” (defined as organ–chemotype combinations) and “genotype” were specified as crossed random effects. *SdEXP* and *SdSAND* were used as additional reference genes in the soft‐normalization models. False‐discovery rate adjusted *P*‐values were calculated for pairwise comparisons of organ–chemotype combinations per ontogenetic stage.

## RESULTS

### Ontogenetic, chemotypic and organ‐specific variation in semi‐polar metabolites in *S. dulcamara*

**Fig. 1:**
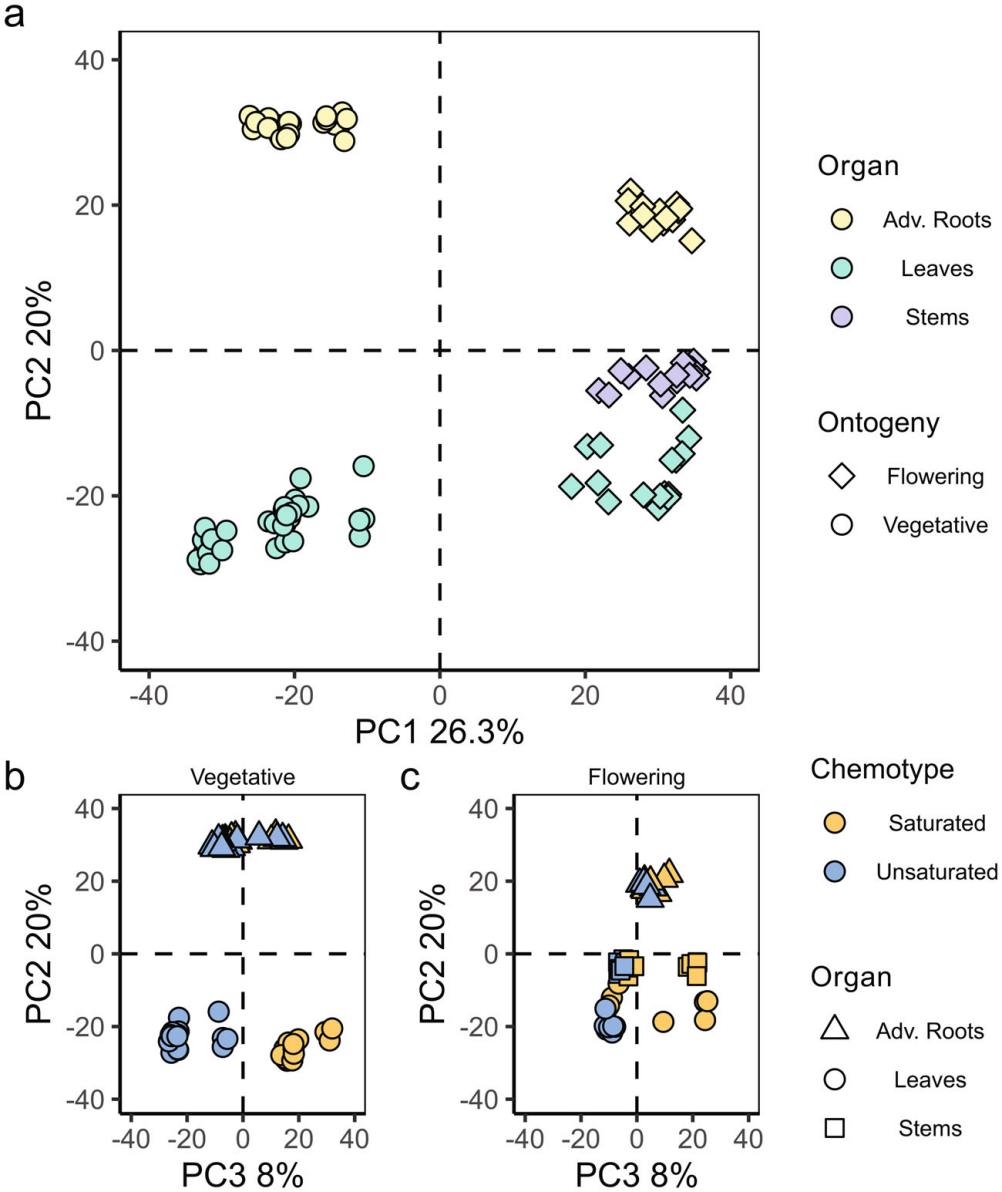
Plot of principal components analysis (PCA) scores based on 2906 features from liquid chromatography–mass spectrometry (LC–MS) eluting in the retention time range 0.75–11.00 min. Symbols represent individual samples. PCA scores (a) of PC1 and PC2. Symbols represent plant ontogeny (diamonds for vegetative, and circles for flowering plants) while symbol colour represents organ from which extracted tissue originated (yellow: adventitious roots, green: leaves, purple: stems). Plots of PC3 and PC2 for vegetative (b) and flowering (c) plants. Symbol shape represents organ‐of‐origin (triangle: adventitious roots, dot: leaves, square: stems), while symbol colour represents leaf chemotype (yellow: saturated steroidal glycosides (SGs), blue: unsaturated SGs). PCA loadings are shown in Figure S1.

To study whether overall phytochemical diversity of semi‐polar metabolites varies among different ontogenetic stages, organs and chemotypes, PCA was performed using 2906 features that eluted in the retention time range 0.75–11.00 min. The samples from vegetative plants clustered together and separated from those of flowering plants on PC1, which explained 26.3% of the observed variance (Fig. 1a; symbols). Additionally, the samples taken from the different organs separated on PC2, which explained 20% of the observed variance (Fig. 1a; colours). Furthermore, leaf samples from the *S*‐ and *U*‐chemotypes in the vegetative stage separated on PC3, which explained 8% of the observed variance (Fig. 1b). This was different in flowering plants: half of the leaf and stem samples of *S*‐chemotype clustered with *U*‐chemotype samples (Fig. 1c). In both vegetative (Fig. 1b) and flowering (Fig. 1c) plants, the root samples of *U*‐ and *S*‐chemotypes clustered close together.

### Mass‐difference networking annotates features associated with chemotypic steroidal glycoside (SG) variation in *S. dulcamara*

**Fig. 2:**
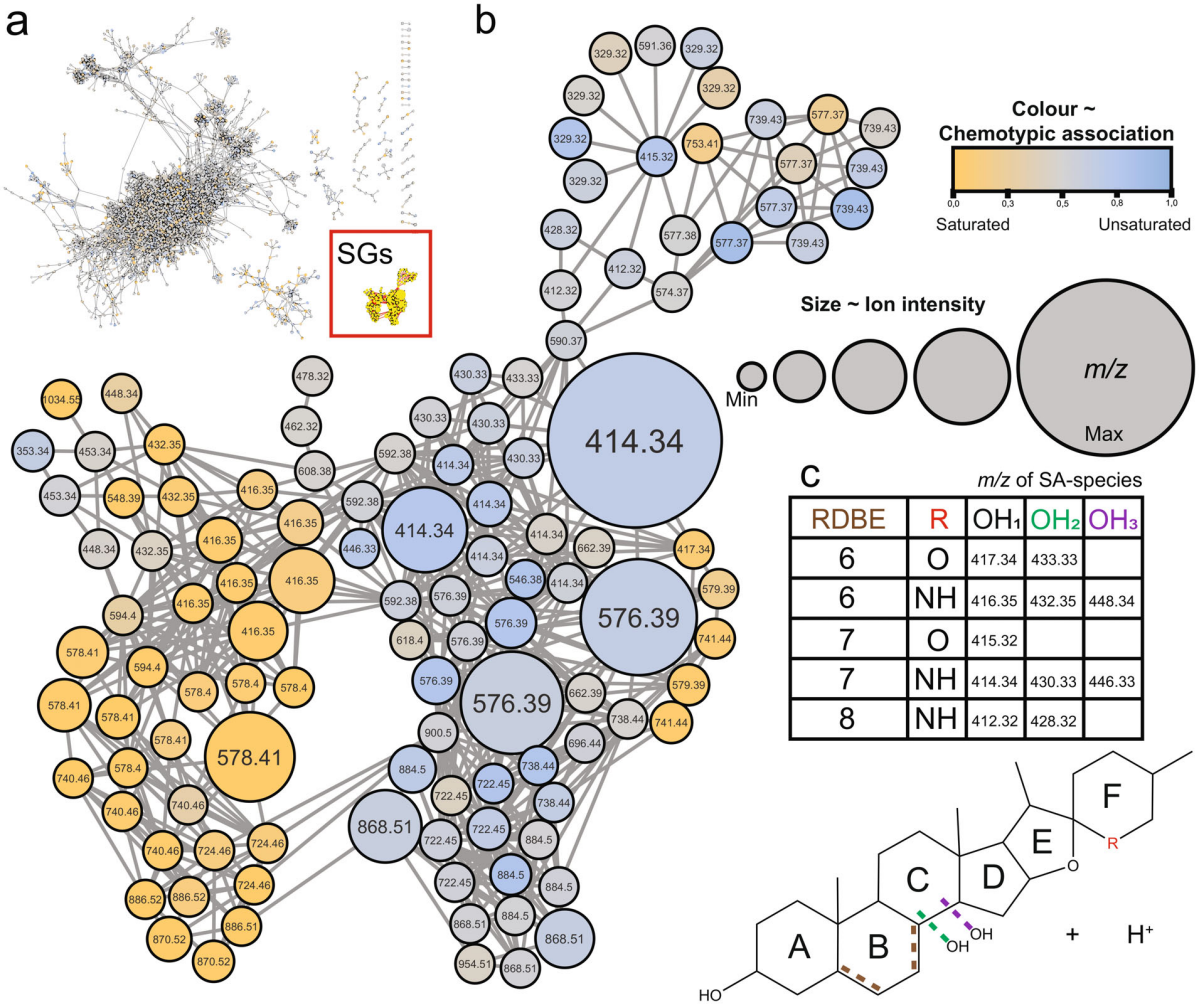
*Ab initio* inferred mass difference network (MDN) of 118 LC–MS features (a), and subnetwork (b) associated with steroidal glycosides, including steroidal glycoalkaloids (SGAs) and steroidal sapogenin glycosides (SSGs). Numbers in nodes depicts mass‐over‐charge (*m/z*) signals resulting from in‐source fragmentation, while connecting edges represent neutral losses (e.g. biotransformations, such as glycosylation, hydroxylation, malonylation, etc.). Node size is proportional to ion intensity in LC–MS. Node colour represents chemotypic association of node (yellow: saturated SGs, blue: unsaturated SGs; grey: neutral). Nodes are based on LC–MS data generated from all samples analysed. Note that isobaric ions in MDN elute at different retention times. (a) Nodes associated with SGs organize in a subcluster which is (b) coloured by chemotypic association of the node. (c) Proposed structural variation of steroidal aglycones based on *m/z* values in the MDN. RDBE, ring‐double bond equivalent; R, NH or O; OH1, 3‐hydroxyl; OH2 and OH3, additional hydroxylation of steroidal aglycones.

To analyse SG chemodiversity among the two *S. dulcamara* SG chemotypes, mass‐difference networks (MDNs) were inferred from LC–MS data of all samples. This approach allows for clustering of features based on specified neutral losses, and for visualization of features as nodes in which the size is proportional to ion‐intensity in the MS (Fig. 2a). Using this approach, features associated with SGs and their in‐source fragments could be classified, since they formed a distinct MDN (Fig. 2a, highlighted SG cluster). Zooming in to this SG‐associated MDN, we visualized which nodes were associated with each of the chemotypes (Fig. 2b, colour gradient).

The SG‐associated network globally separated the *S*‐ (Fig. 2b, yellow circles) and *U*‐ (blue circles) chemotypes. The separation among the two chemotypes was based on nodes that differ in their ring‐double bond equivalent (RBDE; Fig. 2c). Nodes associated with the *S*‐chemotype (yellow) commonly had *m/z* ratios that were 2 Da higher than those associated with the *U*‐chemotype (blue). This indicates that the SGs in *S*‐chemotypes were overall more saturated, as evidenced by a lower RDBE value (Fig. 2c). The grey nodes represent features that are shared among the two chemotypes, and thus form the common metabolome of all detected semi‐polar metabolites (Fig. 2a) and SGs (Fig. 2b) in *S. dulcamara*.

### Mass‐difference networking annotates features associated with structural steroidal aglycone (SA) variation in *S. dulcamara*

To study the structural diversity of the steroidal aglycones in the two *S. dulcamara* chemotypes, the in‐source fragmentation‐based MDNs were further inspected for their mass‐differences. The presence of nodes with both odd and even *m*/*z* values in the MDN suggest that the plants contain both SSG‐ (odd *m/z*) and SGA‐type (even *m*/*z*) SGs (Fig. 2b). Additionally, the MDN shows that steroidal aglycones in both SG classes vary in RDBE levels. Multiple nodes with *m/z* 414.34 and *m/z* 416.35 were detected, associated with the steroidal aglycones of SGAs (steroidal alkamines), and were putatively annotated as solasodine/tomatidenol and soladulcidine/tomatidine (Eich 2008), which have RDBE values of 6 and 7, respectively (Fig. 2c). Two other nodes, with *m/z* 415.32 and *m/z* 417.34, found in top and centre right of the MDN, are associated with the steroidal aglycones of SSGs (steroidal sapogenins), and were putatively annotated as diosgenin/yamogenin and dehydrodiosgenin/dehydroyamogenin (Eich 2008), which have RDBE values of 6 and 7, respectively (Fig. 2c).

Furthermore, the MDN revealed variation in the hydroxylation level of steroidal aglycones in *S. dulcamara* (Fig. 2b). For the putative steroidal alkamine aglycones solasodine (*m/z* 414.34) and soladulcidine (*m/z* 416.35), we found nodes associated with their mono‐hydroxylated (*m/z* 430.33–432.35) and di‐hydroxylated (*m/z* 446.33–448.34; Fig. 2c) analogues, respectively. Interestingly, we detected another pair of SGAs with mono‐hydroxylated (*m/z* 412.32–428.32) steroidal aglycones with an RDBE value of 8 (Fig. 2c). For the putative steroidal saponin e, we found only one pair of nodes (*m/z* 417.34–433.33) indicative of hydroxylation (Fig. 2c). Next to the tabulated *m/z* values (Fig. 2c), two additional nodes, with *m/z* 434.36 (RDBE = 5) and *m/z* 453.34, were annotated as potential SGA‐ and SSG‐type steroidal aglycones, respectively, based on their occurrence in the MDN.

### Mass‐difference networking annotates mass spectra associated with organ‐specific and ontogenetic SG variation in *S. dulcamara*

**Fig. 3:**
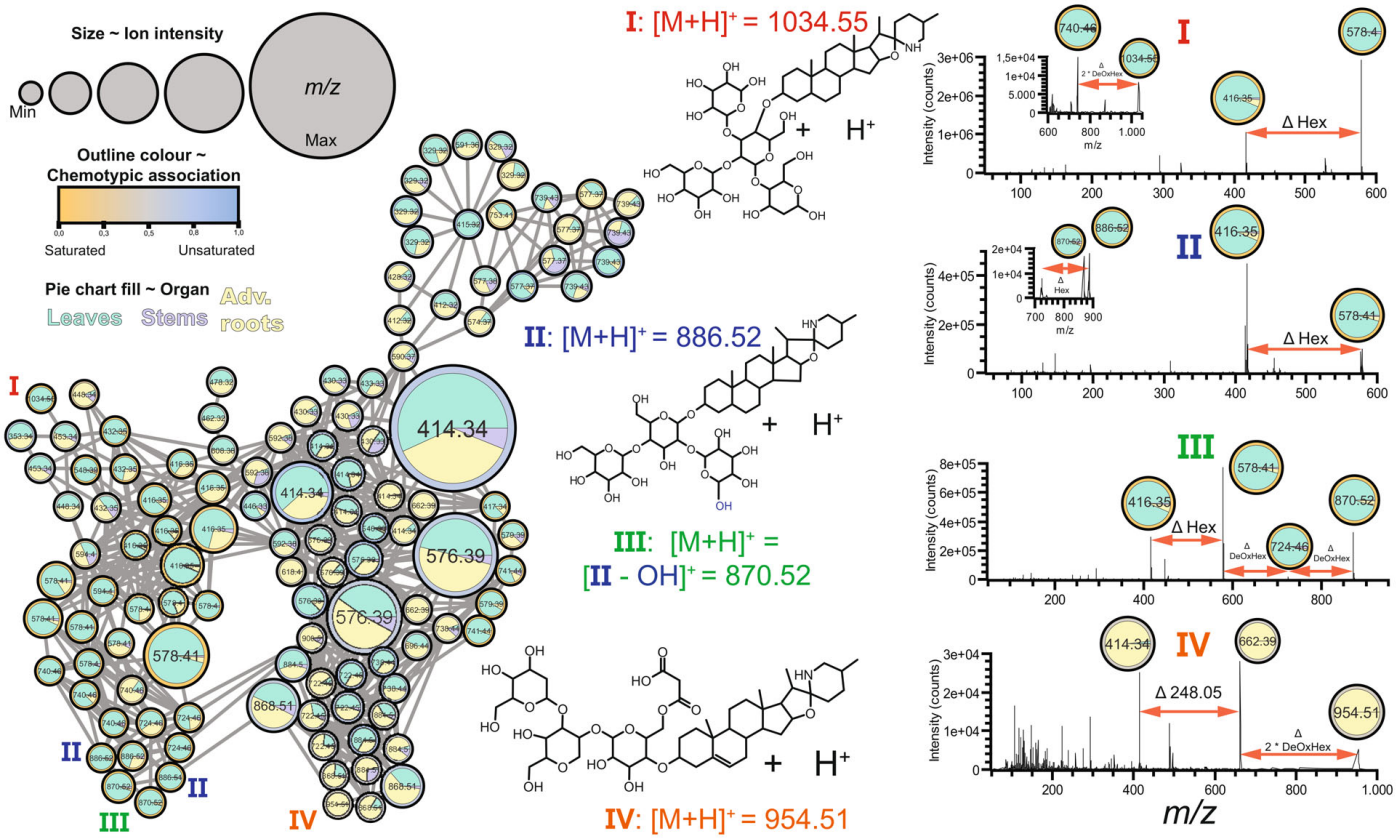
*Ab initio* inferred mass difference networks (MDN) of 118 LC–MS features (nodes) associated with steroidal glycosides, including steroidal glycoalkaloids (SGAs) and steroidal sapogenin glycosides (SSGs). Number in nodes depicts mass‐over‐charge (*m/z*) signal resulting from in‐source fragmentation, while connecting edges represent neutral losses (e.g. biotransformations such as glycosylation, hydroxylation, malonylation, etc.). Node size is proportional to ion intensity in LC–MS. Nodes are coloured by chemotypic association of the node (yellow: saturated SGs, blue: unsaturated SGs; grey: neutral). Relative intensity for every node is visualized as a pie chart coloured by organ (yellow: adventitious roots, green: leaves, purple: stems). Note that isobaric ions in the MDN elute at different retention times. Neutral losses are visualized within mass spectra using red double‐headed arrows. Hex, hexose; DeOxHex, deoxyhexose; Δ 248.05, malonylhexoside. Mass spectra of hypothesized compounds I–VI in this figure are shown in Figure S2, while for compounds V and VI, mass spectra are also shown in Fig. 4.

**Fig. 4:**
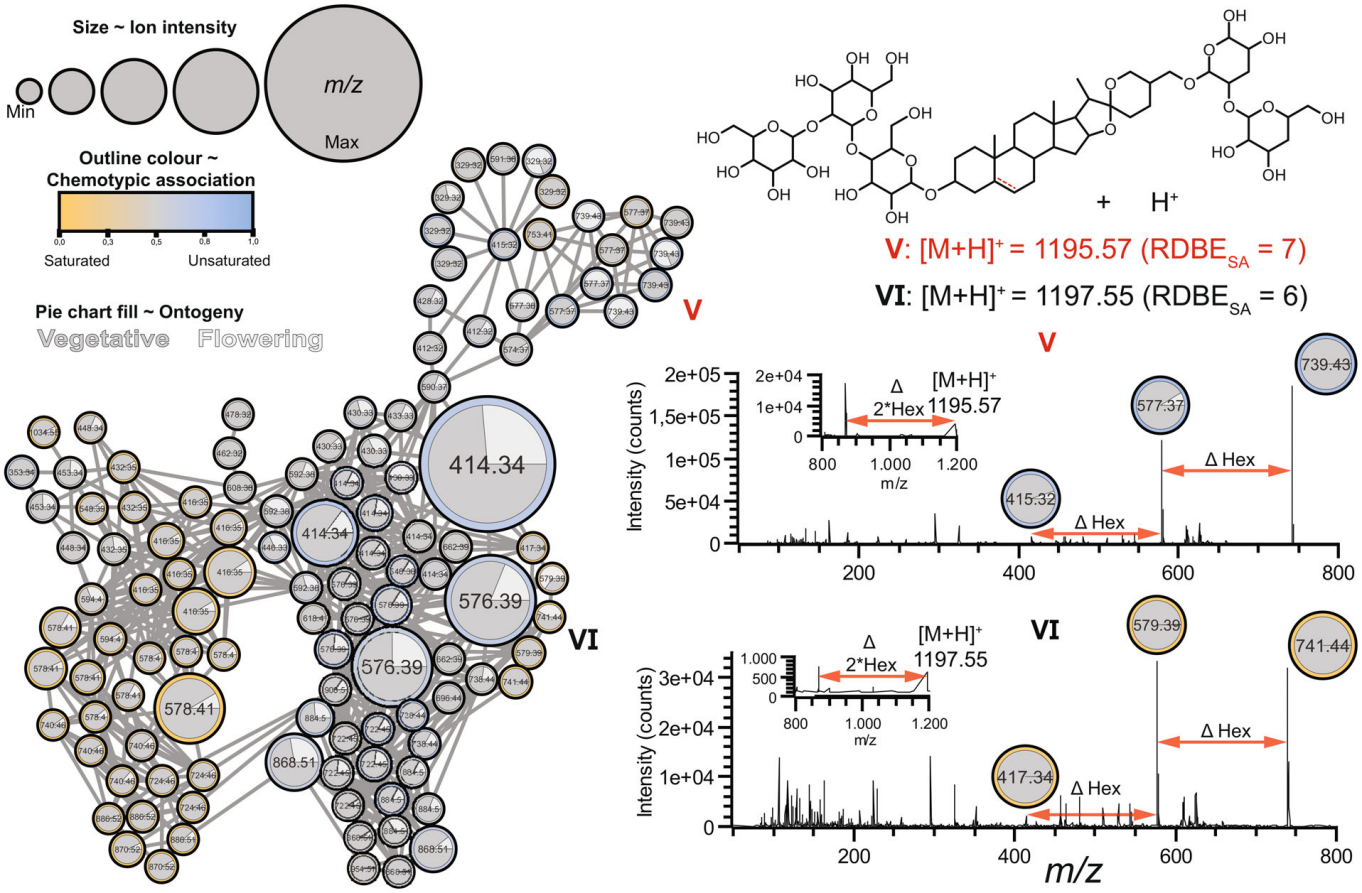
*Ab initio* inferred mass difference networks (MDN) of 118 LC–MS features (nodes) associated with steroidal glycosides, including steroidal glycoalkaloids (SGAs) and steroidal sapogenin glycosides (SSGs). Number in nodes depicts mass‐over‐charge (*m/z*) signals resulting from in‐source fragmentation, while connecting edges represent neutral losses (e.g. biotransformations, such as glycosylation, hydroxylation, malonylation, etc.). Node size is proportional to ion intensity in LC–MS. Node outline and legend network are coloured by chemotypic association of the node (yellow: saturated SGs, blue: unsaturated SGs; grey: neutral). Relative intensity for every node is visualized as a pie chart coloured by ontogeny (dark grey: vegetative, light grey: flowering). Note isobaric ions in MDN elute at different retention times. Neutral losses are visualized within mass spectra using red double‐headed arrows. Hex, hexose; DeOxHex, deoxyhexose. Mass spectra of hypothesized compounds V and VI in this figure are shown in Figure S2, while compounds I‐IV mass spectra are also in Fig. 3.

To study the organ‐ and ontogeny‐specific SG distribution in the two *S. dulcamara* chemotypes, nodes in the MDN were visualized as pie charts showing the relative intensity of the node by organ (Fig. 3) or ontogeny (Fig. 4). Using this visualization method, putative SGs exclusively detected in a chemotype (Fig. 2b), organ‐type (Fig. 3) or ontogenetic stage (Fig. 4) were annotated.

Features associated with saturated SGAs in leaves were the main drivers separating the two clusters of nodes according to chemotype (Figs 2b and 3). A saturated SGA tetraose I (Fig. 3, Figure S2, *m*/*z* values: 416.35, 528.77, 578.40, 740.46 and 1034.55) and two saturated SGA triose II (Fig. 3, Figure S2, *m*/*z* values: 416.35, 454.75, 578.40, 724.46 and 886.52), which are putatively annotated as ‘soladulcine B′ and ‘soladulcine A′, respectively, were detected in the *S* but not in the *U*‐chemotype. Interestingly, the saturated SGAs I and II associated with *S*‐chemotype plants (Figs 2b and 3) were most prominently detected in leaf samples, predominantly in those of vegetative plants (Fig. 4). Furthermore, one saturated SGA triose III (Fig. 3, Figure S2; *m*/*z* values: 416.35, 454.75, 578.40, 724.46 and 870.52) with putative molecular ion *m/z* 870.52 and multiple constitutional isomers were detected, as the molecular ion is found at least twice in the MDN (Fig. 3). Compared to compound II, isomers of III were associated with the substitution of a hexose for a deoxyhexose in the glycoside moiety of III (Fig. 3).

### Malonylglucoside SGA exclusively detected in roots of vegetative *S. dulcamara*

In the MDN coloured by organ‐type, most of the nodes had two or three colours, meaning that they represent features found in leaves, roots and stems. The nodes representing SGs **I**, **II**, **III**, however, were predominantly found in leaves. Interestingly, we found a few nodes that coloured completely yellow, meaning that these features were exclusively detected in roots (Fig. 3). These *m*/*z* features were putatively assigned to a malonylated SGA with molecular ion *m*/*z* 954.51 and base peak *m*/*z* 662.39 (**IV**; Fig. 3). We also found a completely yellow node for *m*/*z* 414.34, which is indicative of a root‐specific unsaturated nitrogenated steroidal aglycone (Fig. 3, Figure S2). The fragmentation pattern of **IV** shows two sequential neutral losses of deoxyhexose (Δ_m/z_ 146.05) moieties, followed by the loss of Δ_m/z_ 248.05 (Fig. 3), the latter of which indicates the loss of a malonylglucoside moiety. This fragmentation pattern, together with the base peak *m*/*z* 662.39, indicates that malonylglucoside is part of a larger trisaccharide moiety that is conjugated to the 3‐hydroxyl position of an unsaturated steroidal aglycone (Fig. 3).

### Steroidal saponin glycosides vary across organs and ontogeny in *S. dulcamara*

A cluster of nodes associated with unsaturated SSGs (**V**) formed a subnetwork on top of the larger MND (Figs 3 and 4). We assigned the *m*/*z* features in these nodes to putative SSG pentosides with molecular ion *m*/*z* 1195.5662 and base peak *m*/*z* 739.4221. Additionally, we detected a node with *m*/*z* 415.31, which is indicative of an unsaturated oxygenated steroidal aglycone (**V**, Fig. 4). The fragmentation pattern of this putative saponin pentoside (Fig. 4, Figure S2) showed sequential neutral losses, indicative of the cleavage of a hexose and a pentose, followed by the loss of three hexoses.

Interestingly, a cluster of five nodes, three of which with *m*/*z* 739.43, were found at the top of the MDN (**V**), suggesting that there are (sub)structural isomers of **V** in *S. dulcamara* (Fig. 4). The colours of the SSG‐associated nodes in Fig. 3 indicate that most features were found in roots and leaves, wherein two nodes (*m/z* 577.37 and 739.43) were more frequently found in stems and roots (Fig. 3; left upper cluster). Interestingly, four out of five nodes with *m*/*z* 739.43 are circled in blue and thus associated with *U*‐chemotype plants, while one node is grey and thus occurs more‐or‐less equally in both chemotypes (Fig. 4). Of the four nodes associated with *U*‐chemotype plants, three were predominantly associated with leaf samples, and one with stem samples (Fig. 3). Nodes in the SSG cluster that were encircled in grey (Fig. 3) were predominantly associated with roots (Fig. 3). Three of the five nodes with *m*/*z* 739.43 are predominantly associated with flowering plants, whereas the other two nodes are mainly detected in vegetative plants (Fig. 4). Additionally, two nodes with *m*/*z* 741.44 (Fig. 4, VI) were found in *S*‐chemotype plants. These *m/z* features were annotated as SGG pentosides, which are the saturated analogues of **V** with putative molecular ion *m*/*z* 1197.55. (Fig. 4).

### Variation partitioning of SG counts using GLM ANOVA simultaneous component analysis (GLM‐ASCA) reveals dynamics of SG variability in *S. dulcamara*

Based on inspection of the EICs of the 12 annotated SA species (Fig. 2c), we counted a total of 3149 scans associated with SGs. To study the variation in SG counts per SA species among chemotypes, organs and ontogenetic stages in the two *S. dulcamara* chemotypes, variation partitioning using a GLM‐based ASCA was performed. The first GLM‐ASCA model describes the interaction between ‘Organ’ and ‘Chemotype’ (Figure S3). Interestingly, SGs with SA species *m/z* 416.35 are associated with latent variables (LV) 1 and 2 for the interaction (Figure S3a), and LV1 for the term ‘Chemotype’. This is in accordance with the chemotype selection, which was done based on *m/z* 416.35 (Figure S3b, c). Furthermore, the LV1 for the term ‘Organ’ (Figure S3a) projects leaf SG profiles in the negative direction (Figure S3b), which is associated with the higher number of SSGs (SA species with *m/z* 415.32, 417.33 and 433.33; Figure S3c) in leaves than in roots or stems. Lastly, LV2 of the term ‘Organ’ (Figure S3a) projects SG profiles of root samples in the positive direction (Figure S3b), which is associated with a higher number of SSGs with SA species *m/z* 433.33 (Figure S3c).

The second GLM‐ASCA model describes the interaction between ‘Sample type’ (different organs per chemotype) and ‘Ontogeny’ (Figure S4). Interestingly, SGs with SA species *m/z* 416.35 are associated with LV1 and LV2 for the interaction and LV1 for the term ‘Chemotype’ (Figure S4a). This is in accordance with the chemotype selection, which was done based on the same *m/z* signal (Figure S4b, c). The LV1 of the interaction term (Figure S4a) separates SG profiles of roots of vegetative and flowering plants (Figure S4b), which is associated with a higher number of SGs with SA species *m/z* 412.32 and *m/z* 433.33 in roots of flowering plants (Figure S4c). In contrast, SSGs with SA species *m/z* 417.33 (Figure S4c) were more present in roots of vegetative plants (Figure S4b). The LV1 for the term ‘Ontogeny’ (Figure S4a), indicated that chemical profiles of samples taken from vegetative and flowering were always clearly separated (Figure S4b). This was related to the higher number of SGs with SA species *m/z* 417.33 and 446.33 (Figure S4c) in vegetative organs, independent of chemotype. Lastly, the LV1 for the term ‘Sample’ (Figure S4a) showed that stems differentiate from the other samples (Figure S4b), which was associated with higher numbers of SGs with SA species *m/z* 416.35, 417.33 and 446.33 (Figure S4c). Furthermore, leaf SG profiles were separated from other organs in the negative direction (Figure S4b), which was associated with higher numbers of SGs with SA species *m/z* 412.32, 415.3 and 433.33 (Figure S4c) in leaves.

### Chemical diversity indices provide insight in SG metabolism across organs and ontogeny in *S. dulcamara*

To compare the levels of SG chemodiversity among organs and across ontogeny in the two chemotypes, we counted the number of glycosylated signals of putative SGs for every SA species annotated by MDN (Fig. 2c). With these data, we calculated the Margalef's richness (D_mg_) and Pielou's evenness (J) for every sample. Furthermore, we used the total ion current TIC_SG_ as a proxy for total SG quantity and studied its relationship with SG chemodiversity indices D_mg_ and J in different organs across ontogeny.

**Fig. 5:**
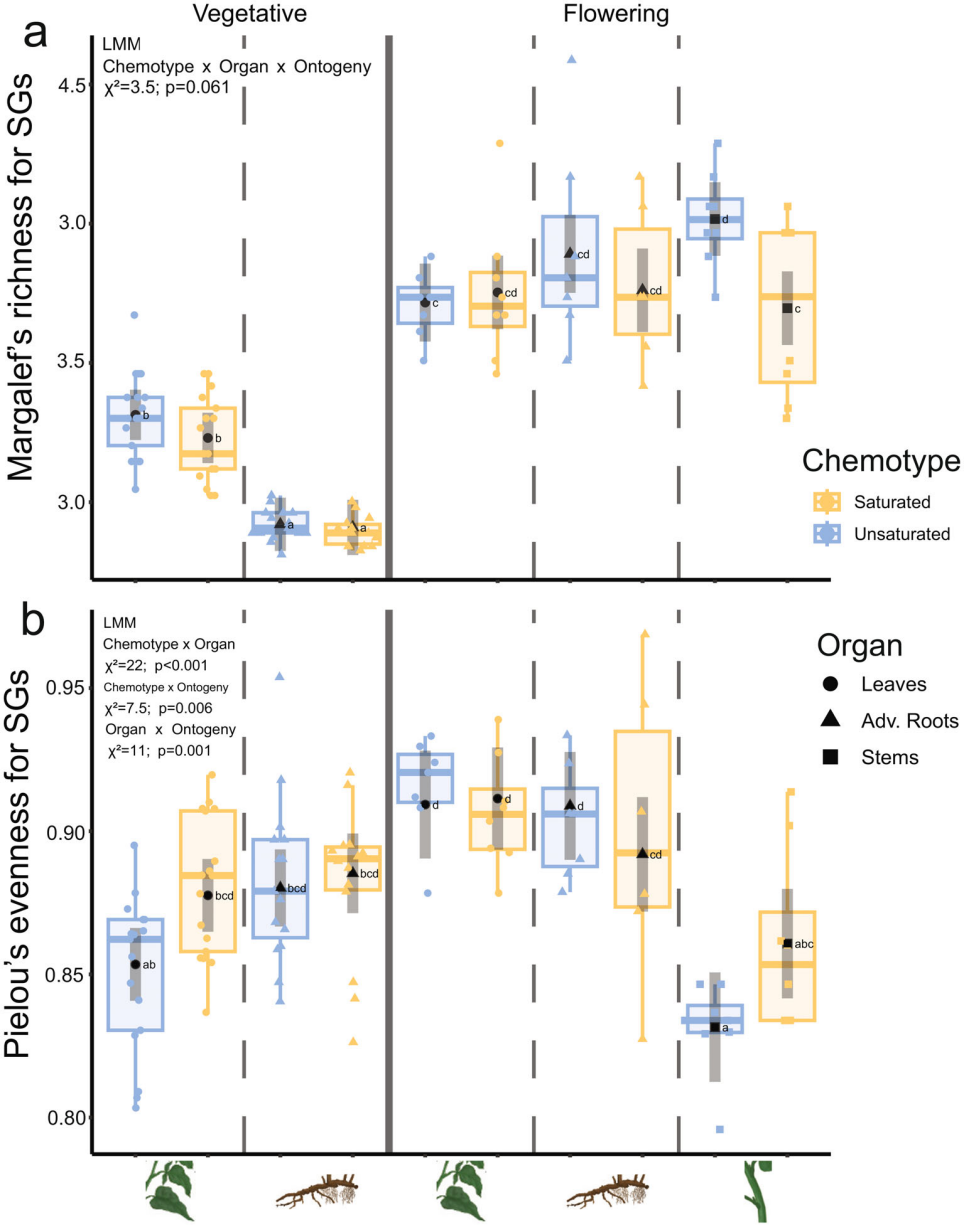
Diversity indices based on a total of 3149 counted steroidal glycosides (SG) of 12 steroidal aglycone (SA) species. (a) Margalef's richness and (b) Pielou's evenness for SGs based on SG counts for 12 annotated SA species selected from mass‐difference networking. Estimated marginal means (black symbols) and 95% confidence intervals (dark grey lines). Symbol shape represents organ (triangle: adventitious roots, dot: leaves, square: stems). Raw data are summarized as boxplots and individual data points are plotted for vegetative (left from thick line) and flowering (right) plants. Linear mixed models (LMMs) were built with the three‐way interaction ‘chemotype’, ‘organ’, and ‘ontogeny’ as fixed effects, and plant individuals nested within genotype as random effect. For SG evenness, the three‐way interaction term was removed from the model because it was not significant. Letters show outcome of Tukey's post‐hoc test on estimated marginal means, and groups sharing a letter were not significantly different. Wald's *χ* ^2^‐test (Table S2) was used to test for interactions between explanatory variables. *P* < 0.1; *P < 0.05; ***P < 0.001.

In general, the median SG richness was higher in samples of flowering (D_EMM_ > 3.5) than in those of vegetative (D_EMM_ < 3.5; Fig. 5a) plants. In vegetative plants, SG richness was higher in leaves (*S*‐chemotype: D_EMM_ = 3.23 ± 0.0455; 95% CI: [3.14–3.32]; *U*‐chemotype: D_EMM_ = 3.31 ± 0.0455; 95% CI: [3.22–3.40]) than in roots (*S*‐chemotype: D_EMM_ = 2.91 ± 0.0499; 95% CI: [2.81–3.01]; *U*‐chemotype: D_EMM_ = 2.92 ± 0.0482; 95% CI: [2.82–3.02]; Fig. 5a). In flowering plants of the *U*‐chemotype, SG richness values were lower for leaves (D_EMM_ = 3.72 ± 0.0705; 95% CI: [3.58–3.86]) than for stems (D_EMM_ = 4.02 ± 0.0665; 95‐% CI: [3.88–4.15]; Fig. 5a). Furthermore, stems of flowering plants of the *U*‐chemotype have higher predicted SG richness than those of the *S*‐chemotype (D_EMM_ = 3.70 ± 0.0665; 95% CI: [3.56–3.83]; Fig. 5a).

Next to SG richness, we also analysed SG evenness. The measures of SG evenness varied more across flowering (EMMs J_min‐max_ = 0.832–0.911; Fig. 5b, right) than vegetative (EMMs J_min‐max_ = 0.853–0.885; Fig. 5b, left) organs, with a maximum observed EMM of J_max_ = 0.911 in leaves of flowering plants (Fig. 5b, right). In vegetative plants, SG evenness tended to be higher in *S*‐chemotypes than in *U*‐chemotype for both leaves (*S*‐chemotype J_EMM_ = 0.878 ± 0.00641; 95% CI: [0.865–0.890]; *U*‐chemotype J_EMM_ = 0.853 ± 0.00641; 95% CI: [0.841–0.866]) and roots (*S*‐chemotype J_EMM_ = 0.885 ± 0.00701; 95% CI: [0.871–0.899]; *U*‐chemotype J_EMM_ = 0.880 ± 0.00679; 95% CI: [0.867–0.894]), but the differences were not significant (Fig. 5b). In flowering plants, predicted leaf SG evenness is higher (*S*‐chemotype J_EMM_ = 0.911 ± 0.00899; 95% CI: [0.894–0.929]; *U*‐chemotype J_EMM_ = 0.909 ± 0.00899; 95% CI: [0.890–0.928]) than that of stems (*S*‐chemotype J_EMM_ = 0.861 ± 0.00964; 95% CI: [0.842–0.880]; *U*‐chemotype J_EMM_ = 0.832 ± 0.00964; 95% CI: [0.812–0.851]), but not than that of roots (*S*‐chemotype J_EMM_ = 0.892 ± 0.01007; 95% CI: [0.872–0.912]; *U*‐chemotype J_EMM_ = 0.909 ± 0.00949; 95% CI: [0.890–0.928]; Fig. 5b). Taken together, this means that both organ and ontogeny interactively impact variation in the measures of chemodiversity.

Additionally, we analysed the relationship between SG diversity indices D_mg_ and J with TIC_SG_. Overall D_mg_ correlated negatively with TIC_SG_ (R = − 0.73, *P* = 0.017; Figure S7a). Specifically, in flowering *S*‐chemotype plants, D_mg_ correlated negatively with the TIC_SG_ in leaves (R_L_ = −0.82, *P* = 0.012) and stems (R_S_ = −0.77, *P =* 0.024; Figure S7b). Overall *J* did not correlate with TIC_SG_ (R = 0.12, *P* = 0.74; Figure S7c). Furthermore, *J* correlated positively with the TIC_SG_ in leaves of *S*‐chemotype (R_L_ = 0.8, *P* = 0.017) and stems of *U*‐chemotype (R_S_ = 0.76, *P* = 0.028) plants in the flowering stage (Figure S7d).

### Expression patterns of candidate *GAME* genes explain SG diversification among organs across ontogeny

**Fig. 6:**
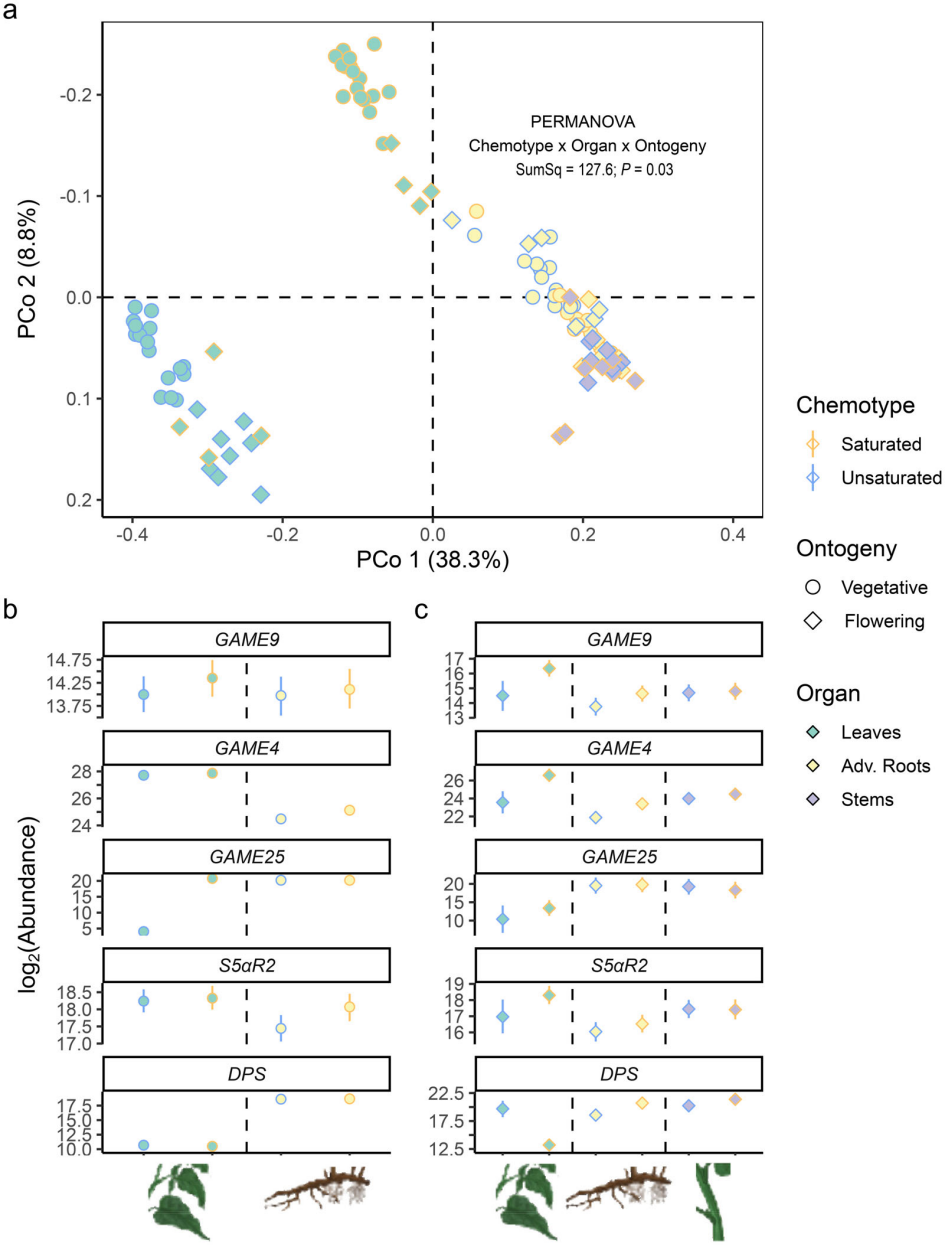
The log_2_‐transformed transcript abundances from reverse transcription–quantitative polymerase chain reaction (RT‐qPCR) analyses for (a) naïve and (b, c) soft‐normalization models based on five candidate steroidal glycoside (SG) metabolism genes and two reference genes. (a) Principal coordinates analysis of log_2_‐transformed transcript abundances from the naïve model. Symbol shape represent ontogeny (diamonds: vegetative plants; asterisks: flowering plants); fill colour and line type represent organ‐of‐origin (light yellow triangles: adventitious roots, green dots: leaves, purple squares: stems). (b, c) Estimated marginal means (black symbols) and 95% credible intervals (coloured lines). Colours represent leaf chemotypes (blue: unsaturated steroidal glycosides (SGs); yellow: saturated SGs. Symbol shape represents ontogeny (triangle: adventitious roots, dot: leaves, square: stems) while line colour represents leaf chemotype (yellow: saturated SGs, blue: unsaturated SGs). Different organs are separated by a dashed line. A soft‐normalization model was built using *EXP* and SAND as reference genes (priors) providing informed posteriors for target genes *GAME9*, *GAME4*, *GAME25*, *S5αR2* and *DPS*. (b) vegetative plants (c) flowering plants.

To investigate the potential chemotype‐ and organ‐specific expression for selected genes, RT‐qPCR analyses were performed with total RNA extracted from different organs of vegetative and flowering *S. dulcamara* individuals. Primers were designed using a homology‐based approach with previously characterized *GAME* genes from closely related cultivated *Solanum* spp. Transcript abundances (counts) were calculated based on C_q_ values given the primer efficiencies of candidate and reference genes. First, PCA and PERMANOVA were performed on a naïve model (Fig. 6a). The expression levels of *U*‐chemotype leaf samples separated from all other samples on PCo1, which explained 38.4% of the observed variation (Fig. 6a). Analogously, the separation of *S*‐chemotype leaf samples from stem and root samples was associated with PCo2, which explained 8.8% of the observed variation (Fig. 6a). Furthermore, leaf samples were organized in subclusters based on ontogeny (symbols; Fig. 6a).

Second, we analysed transcript abundances for every candidate gene based on an informed model which considers *EXP* and *SAND* as reference genes (Fig. 6b, c). *GAME9* codes for a transcription factor associated with steroidal glycoalkaloid biosynthesis (Cárdenas *et al*. 2016; Nakayasu *et al*. 2018). *SdGAME9* transcript abundance did not differ across organ or chemotype, neither in vegetative plants (Fig. 6b, *SdGAME9*) nor in flowering plants (Fig. 6c, *SdGAME9*). It is noteworthy that the expression pattern of *SdGAME9* in different organs, though not significantly different, closely resembles that of *SdGAME4* and *SdS5αR2* in flowering (Fig. 6b), but not in vegetative (Fig. 6a), plants.

*GAME4* codes for a cytochrome P450 enzyme that is involved in early oxidation of steroidal precursors towards SGA biosynthesis (Itkin *et al*. 2013; Paudel *et al*. 2017). In vegetative plants, *SdGAME4* transcript abundances were higher in leaves than in roots. Additionally, *S*‐chemotype roots had 1.37‐fold higher transcript abundance than *U*‐chemotype roots (*P* = 0.0062, Fig. 6b). When flowering, *SdGAME4* transcript abundances in leaves and roots of *S*‐chemotype plants were 1.24‐fold (*P =* 0.0039) and 2.9‐fold (*P =* 0.0094) higher, respectively, than in leaves and roots of *U*‐chemotype plants (Fig. 6c).

*GAME25* and *S5αR2* are two genes associated with double bond reduction in other *Solanum* spp. They code for a short‐chain dehydrogenase/reductase (Lee *et al*. 2019; Sonawane *et al*. 2018) and a steroid Ç5α‐reductase (Akiyama *et al*. 2019), respectively. GAME25 catalyses the first dedicated step towards saturated SGs. In leaves of vegetative *U*‐chemotype plants, *SdGAME25* abundance is significantly lower, in fact close to the detection limit, compared to leaves of *S*‐chemotype plants (Fig. 6b, *SdGAME25*). In contrast, *SdGAME25* transcript abundances are not significantly different among leaves of the two chemotypes when plants are flowering (Fig. 6c, *SdGAME25*). S5αR2 is the second enzyme involved in the reduction of the double bond in the B‐ring of steroidal glycosides in *Solanum* spp. Neither in leaves nor in roots of vegetative plants did we find statistically significant chemotypic differences in *SdS5αR2* abundance, although the expression levels in roots of *S*‐chemotypes were visibly higher than those in *U*‐chemotypes (Fig. 6b, *SdS5αR2*). In flowering plants, leaves of *S*‐chemotype plants had higher *SdS5αR2* transcript abundances than leaves of *U*‐chemotype plants, but the difference was not significant (Fig. 6c).

DPS is a 2‐oxoglutarate‐dependent dioxygenase that catalyses spirostanes into solanidanes in *S. tuberosum*. DPS catalyses the first dedicated step towards solanidanes by downstream C16α‐hydroxylation. In vegetative plants, *SdDPS* abundance is higher in roots (>2^17^) than leaves (<2^13^; Fig. 6b). In flowering plants, *SdDPS* abundance was the lowest in *S*‐chemotype leaves (<2^15^), much lower than in all other organs (>2^17.5^; Fig. 6c). In contrast, the absolute expression levels were 4.47‐fold higher in roots of *S*‐chemotypes than in *U*‐chemotype plants (*P* < 0.01; Fig. 6c, *SdDPS*).

## DISCUSSION

To date, SG variation in *S. dulcamara* leaves (Calf *et al*. 2018) and roots (Chiocchio *et al*. 2023) has been described using metabolomic approaches, but the extent of other levels of intra‐individual variation in SG chemodiversity in *S. dulcamara* chemotypes remained unknown. Here, we show that there are additional levels of organ‐ and ontogeny‐specific variation in SG chemodiversity in two studied *S. dulcamara* leaf chemotypes. Our untargeted metabolomic approached yielded 2906 picked LC–MS features. Combining PCA with mass‐difference networking resulted in 118 SG‐associated features. This allowed us to investigate intraspecific SG metabolism and to postulate the presence of at least 12 SA species in the *S. dulcamara* extracts. These analyses revealed that leaves of vegetative individuals of the two selected chemotypes have very distinct SG profiles, whereas roots on vegetative and flowering plants do not. On flowering plants, the SG profiles of leaves and stems were less distinct, showing that chemodiversity is affected by ontogeny. Finally, we have explored SG chemodiversity through manual investigation of EICs of the 12 annotated SA species. We counted 3149 mass spectra associated with SGs. These SG counts were used for variation partitioning using GLM‐ASCA and to calculate the chemical diversity indices $D_{\mathrm{mg}}$ and J, which were used for univariate analyses using L(M)Ms. These analyses showed that overall chemodiversity varies among organs and over ontogeny, but not as much among chemotypes. Lastly, we used a homology‐based approach to select five candidate and two reference genes of which the expression was measured using RT‐qPCRs. These analyses showed that gene expression patterns of enzymes involved in SG biosynthesis were in line with the observed SG profiles. Our experiments show that organ‐ and ontogeny‐specific variation in SG chemodiversity relates to the expression of candidate genes in SG metabolism.

### Organ and ontogenetic variation in SG chemodiversity

Chemical profiling of the organs of SG chemotypes across ontogeny followed by MDN revealed that SG diversity in *S. dulcamara* is broader than previously described (Calf *et al*. 2018; Chiocchio *et al*. 2023). As expected, the putatively annotated saturated soladulcine B (**I**), and soladulcine A (**II**), are both predominantly detected in leaves of the *S*‐chemotype (Lee *et al*. 1994).^1^ Unsaturated analogues of these SGAs were found in leaves of the *U*‐chemotype, but also in roots of both chemotypes. In this study, unsaturated SSGs such as **V** were detected at high levels in the leaves and the roots of *U*‐chemotype plants. Interestingly, these compounds were also detected at high levels in roots of *S*‐chemotype plants, whereas they were very low in their leaves. This implies that chemotypic differentiation is leaf‐specific and not associated with the absence or presence of genes coding for SG biosynthesis. Furthermore, soladulcine B is a stereoisomer of α‐tomatine, which suggests that soladulcine B may have similar herbivore‐deterrent properties as these described for α‐tomatine (Bailly 2021; You & van Kan 2021). In terms of intra‐individual organ‐specific variation, we detected a malonylglucoside SGA (**IV**) that was exclusively found in roots. Modifications like glycosylation and malonylation may increase the polarity of SGs and thereby their transportability, storability, and biological activity (Wolters *et al*. 2023). Malonylation is a process that is described for diterpene glycosides, a class of defence compounds that are known in other Solanaceae such as *Nicotiana attenuata* and *Capsicum* spp. (Heiling *et al*. 2010; Macel *et al*. 2019). Furthermore, specific decorations of 17‐hydroxy‐geranyl linalool by malonyl‐ and glycosylation are shown to solve the autotoxicity problem of diterpene‐based defences in *N. attenuata* (Heiling *et al*. 2021). In addition, malonylated compounds may be more suitable for exudation into the rhizosphere, as root exudates are mostly polar, water‐soluble compounds (Van Dam & Bouwmeester 2016). Lastly, one of the saturated SSGs (**VI**), was detected in flowering, but not in vegetative aboveground organs of *S*‐chemotype plants, which suggests that this compound (class) may have a specific function in the interactions with pollinators. Further experiments are needed to infer the ecological functions of the various SGs in *S. dulcamara*.

We counted SGs for every annotated SA species. These SG counts allowed us to perform variation partitioning, thereby revealing the dynamics of SG chemodiversity among chemotypes and organs, and across ontogeny. These GLM‐ASCA analyses corroborated the conclusions of the molecular network analyses. First, they confirmed that *m/z* 416.35, which was used to chemotype plants before chemical profiling, was clearly associated with chemotypic differences. Furthermore, we found that the numbers of SSGs with SA species *m/z* 415.32, 417.33 and 433.33 were higher in leaves than other organs, which we did not anticipate from results of previous studies (Calf *et al*. 2018; Chiocchio *et al*. 2023). In these previous studies, plants were sampled during the vegetative stage, which might explain why SSGs such as **V** and **VI** were previously not detected. In addition, our analyses showed that SG chemodiversity can also significantly vary across ontogeny. In particular, ontogenetic differentiation may also occur in roots, as evidenced by LV1 of the interaction term in the GLM‐ASCA model that partitioned the variation between sample type and plant ontogeny. In addition to variation partitioning, the SG counts were also used to calculate SG richness and evenness, which served to compare SG chemodiversity in different chemotype–organ combinations. Interestingly, SG richness was higher in flowering than vegetative plants. This is in line with the common view that SGs are constitutive chemical defences to protect flowering and fruiting plants from leaf, and potential fitness, loss (Paudel *et al*. 2017; Panda *et al*. 2022). In vegetative plants, SG richness is significantly higher in leaves than in roots, while the opposite trend is observed in flowering plants. Considering that *S. dulcamara* is a perennial with overwintering roots, allocating more metabolites to the root may reflect patterns of optimal defence allocation (De Jong & Van Der 2000; van Dam & van der Meijden 2018). As genes related to the biosynthesis of SGs are expressed in both roots and shoots in both flowering and vegetative plants, differences in root and shoot chemodiversity may be related to differences in transport dynamics. For glucosinolates, the expression of specific transporters in shoots and roots of plants are important drivers of differences in root and shoot glucosinolate profiles (Nour‐Eldin *et al*. 2017). Whether similar mechanisms are regulating SG allocation in *S. dulcamara* has yet to be determined.

### Chemodiveristy of SG in relation to expression of candidate *GAME* genes in *S. dulcamara*

We found that leaf SG chemotypes are not expressed similarly in roots of vegetative plants at the level of both SGs and transcripts. Surprisingly, when flowering, half of the *S*‐chemotype plants showed SG profiles characteristic of *U*‐chemotype plants in aboveground organs. We found that the *S*‐chemotype plants which clustered with *U*‐chemotype plants in the multivariate analyses also had lower expression levels of *SdGAME25* in their leaves compared to plants that retained a SG profile characteristic of *S*‐chemotype plants. GAME25 catalyses the conversion of unsaturated into saturated SGs (Lee *et al*. 2019; Sonawane *et al*. 2018) and hence it was suggested that allelic variation in the *S*‐chemotype would be the cause of the chemotypic differences between *S. dulcamara* leaves (Calf 2019). However, since the roots of *S*‐chemotype plants also express *SdGAME25*, there must be another level at which this leaf chemical polymorphism is maintained. Furthermore, the relatively high expression levels of *SdGAME25* in leaves in the flowering *S*‐chemotype correlates with the presence of saturated analogues of unsaturated SSGs, such as **V**. Sonawane *et al*. (2018) showed that overexpression of *GAME25* in eggplant not only increases saturated SGA production, but also results in the increased production of saturated SSGs. The concurrent increase of saturated SGAs and SSGs in leaves of the *S*‐chemotype thus strongly suggests that *SdGAME25* plays an important role in maintaining SG chemodiversity at different times during the ontogeny of *S. dulcamara*.

The high levels of (unsaturated) SGs in leaves of the *U*‐chemotype can be explained by differential expression of *SdGAME4* across chemotypes. When flowering, *SdGAME4* expression is lower in leaves of *U*‐ than *S*‐chemotype plants. The enzyme coded for by *GAME4* catalyses the first dedicated step in SGA production. Therefore, a higher expression level of *SdGAME4* likely drives SG biosynthesis towards SGA biosynthesis, thereby downregulating SSG accumulation (Paudel *et al*. 2017). Indeed, RNA interference‐mediated silencing of *SdGAME4* decreased SGA, and increased SSG accumulation in *S. lycopersicum* (Itkin *et al*. 2013), and *S. tuberosum* (Paudel *et al*. 2017). This is in line with our observation that roots of *U*‐chemotype plants show lower expression levels of *SdGAME4* and higher abundance of SSG. The low *SdGAME4* may have redirected steroidal precursors into the SSG branch of the SG biosynthetic pathway. Furthermore, the relatively low *SdGAME4* expression in roots compared to leaves, explains why we previously found many SSGs in roots (Chiocchio *et al*. 2023), but not as many in leaves (Calf *et al*. 2018).

The enzyme encoded by *DPS* converts spirostanes into solanidanes in *S. tuberosum* through an initial C16α hydroxylation. Hydroxylation reactions of the steroidal aglycone lead to additional hydroxyl groups (Sonawane *et al*. 2022). These can potentially be glycosylated, thereby generating bidesmodic steroids, such as the putative compounds **V** and **VI** in *S. dulcamara*. In the case where the product of *SdDPS* has similar catalytical activity as that of *StDPS* (Akiyama *et al*. 2021), then the differential expression of *SdDPS* in roots of *U*‐ and *S*‐chemotypes may cause an additional level of SG diversity. In leaves of flowering plants, *SdDPS* expression is lower in *S*‐chemotype than *U*‐chemotype plants, while the opposite is observed for *SdGAME4* and *SdGAME9*. Specifically, *SdDPS* abundance is increased in leaves of flowering *U*‐chemotype plants compare to vegetative plants. Taken together, this suggests that there might be a trade‐off between expression of *SdGAME4* and *SdGAME9* on the one hand, and *SdDPS* on the other hand. Recently, other 2‐oxoglutarate‐dependent dioxygenases, such as GAME33 and GAME34, were associated with expansion of steroidal alkaloid structural diversity in *S. lycopersicum* and *S. habrochaites*, respectively (Sonawane *et al*. 2022). It is likely that 2‐oxoglutarate‐dependent dioxygenases have also driven expansion of SG chemodiversity in *S. dulcamara*. Lastly, the congruence among the expression patterns of different genes, in particular *SdGAME25*, *SdGAME9*, *SdGAME4* and *SdS5αR2*, suggests that these genes are co‐regulated. Indeed, in tomato and potato genes related to SSG and SGA biosynthesis are organized into metabolic gene clusters (Cárdenas *et al*. 2015), which may be co‐regulated by common transcription factors. Further studies, for example using gene‐edited plants lacking one or more of these genes, are needed to fully understand the regulatory mechanisms of these different layers of intra‐individual chemodiversity.

### Implications for chemodiversity research

In conclusion, our analyses provide new insights into the extent and regulation of intraspecific and intra‐individual SG chemodiversity in two *S. dulcamara* chemotypes by combining metabolic analyses with expression analyses of genes involved in the biosynthesis of SGs. Gene expression analyses associated transcripts abundances of candidate genes in SG metabolism with SG chemodiversity. The expression patterns of *SdGAME4*, *SdGAME25* and *SdDPS* were linked to chemotype‐, organ‐ and ontogeny‐specific intra‐individual variation in SG chemodiversity. We used a homology‐based approach for the gene expression analyses, assuming that these gene structure and functions are conserved among related *Solanum* spp. However, functional genetic analyses are needed to show that candidate genes are indeed the casual agents of SG chemodiversity among organs, ontogeny and chemotypes of *S. dulcamara*. Although the combination of MDN, chemical diversity and gene expression analyses provides new insights into the regulation of intra‐individual SG chemodiversity in two *S. dulcamara* chemotypes, we realize that the current approach of quantifying SG chemodiversity in terms of richness and evenness may underestimate the total extent of structural SG diversity in *S. dulcamara*. Additional MS‐based studies investigating structural SG chemodiversity in *S. dulcamara* would benefit from a tandem MS approach, allowing for spectral–database (Wang 2017) and compound–database (Dührkop *et al*. 2019) based dereplication and subsequent propagation of annotations (Ernst *et al*. 2019; Quinlan *et al*. 2022). Alternatively, NMR‐based metabolomics approaches may provide complementary insights into structural SG diversity, especially for chemical evenness, as peak intensities in NMR are directly proportional to concentration of the metabolite. Since our work focused on chemical diversity, we controlled for the effect of genetic diversity by studying clonally propagated plants from selected genotypes of known leaf chemotypes. To study whether differential regulation of *SdGAME25* across ontogeny indeed regulates SG chemodiversity, an experiment with plants grown from seed, rather than cuttings, would need to be performed. Combined with segregation pattern analysis of the chemotype, such an approach allows testing whether differential regulation of *SdGAME25* is controlled by a single locus across ontogeny in *S. dulcamara*. Considering that SG chemotype is heritable (Calf 2019) and related to differences in leaf herbivore and pathogen resistance (Calf *et al*. 2018, 2019; Sonawane *et al*. 2018; Wolters *et al*. 2023), our findings suggest that the existing intraspecific diversity in *S. dulcamara* may have resulted from differential selection pressures exerted by biotic interactors. In addition, the observed intra‐individual chemodiversity suggests that aboveground and belowground chemodiversity may be regulated and selected for independently. We also found that over the course of ontogeny, different types of SG become more prominent, which may be an indication that other interactions, e.g. with pollinators, may be prioritized when plants are flowering. Our work highlights that phytochemical variation among organs and across ontogeny are important dimensions of chemodiversity that need to be considered in chemodiversity experiments. We hypothesize that such processes increase phytochemical dissimilarity in *S. dulcamara* populations, which, in turn, may increase individual plant fitness under field conditions.

## AUTHOR CONTRIBUTIONS

RAA and NMvD conceived and designed the study. NMvD prepared the F1 plants. RAA, IC, and BS performed greenhouse experiments. RAA and IC conducted phytochemical analyses using the method of FV. BS and RS performed gene expression. RAA and IC analysed LC–MS data. IC extracted, validated and counted steroidal glycosides. RAA applied mass‐difference networking, built models and prepared figures. RAA wrote a draft manuscript with feedback from NMvD and FV. All authors provided feedback and agreed the final manuscript.

## FUNDING INFORMATION

RAA, RS, FV and NMvD gratefully acknowledge the German Research Foundation (DFG) for funding to the Research Group ChemDiv (DFG‐FOR 3000/1, P4 DA 1201/10‐1), iDiv (DFG‐FZT 118, 202548816) and ChemBioSys (DFG‐SFB 1127, 239748522). IC was funded by a grant from the Deutscher Akademischer Austauschdienst (Short‐Term grant, 2021 Number 57552336) and BS was funded by an ERAMSUS+ grant from the European Union.

## CONFLICT OF INTEREST

The authors declare no competing interests.

## ETHICS STATEMENT

There were no studies with human and/or animal participants.

## Supporting information


**Data S1.** Supporting Information.

**Figure S1.** (a) Scree and (b, c) loadings plots of principal components analysis (PCA) of features (dots) from liquid‐ coupled to mass spectrometry (LC–MS).

**Figure S2.** Mass spectra of steroidal glycosides that vary across leaf chemotypes (I, II, III), organ type (IV) and ontogeny (steroidal saponin glycosides; V and VI).

**Figure S3.** Generalized linear model (GLM) based anova–simultaneous component analysis (ASCA) of 3149 counted steroidal glycoside (SG) based on 12 steroidal aglycone (SA) species selected from mass‐difference networking. GLM was modelled with the interaction between chemotype and tissue as fixed effect. (a) Scree plot showing latent variables (LV) and their explained variances. (b) Latent variables for interaction terms (LV1 and LV2), chemotype (LV1) and tissue (LV1 and LV2). Symbol shape represents organ‐of‐origin (triangle: adventitious roots, dot: leaves, square: stems) while symbol colour represents leaf chemotype (yellow: saturated SGs, blue: unsaturated SGs). (c) Loading bar plots showing relative importance of SA species in separation of treatments in the latent variables plot. Bar colours represent nature of the SA species (steroidal glycoalkaloids (SGA): orange; steroidal saponin glycosides (SSG): purple), bar colour shade is based on ring double bond equivalent (RDBE) of SA species.

**Figure S4.** Generalized linear model (GLM) based anova–simultaneous component analysis (ASCA) of 3149 counted steroidal glycoside (SG) based on 12 steroidal aglycone (SA) species selected from mass‐difference networking. GLM modelled with interaction between sample type and ontogeny as fixed effect. (a) Scree plot showing latent variables (LV) and their explained variances. (b) Latent variables for interactions term (LV1 and LV2), chemotype (LV1) and tissue (LV1 and LV2). Symbol shape represents ontogeny (diamonds: vegetative plants; asterisks: flowering plants). (c) Loading bar plots showing the relative importance of SA species in the separation of treatments in the latent variables plot. Bar colours represent nature of the SA species (steroidal glycoalkaloids (SGA): orange; steroidal saponin glycosides (SSG): purple), while bar shade is based on the ring double bond equivalent (RDBE) of the SA species.

**Figure S5.** Validation of GLM–ASCA model with interaction between chemotype and organ as fixed effect, showing (a) significance of model terms; (b) univariate model fit (pseudo‐*R* ^2^) for every model variable; and (c) variable importance (Vector Norm) for every model variable. Only responsive variables with higher pseudo‐*R* ^2^ in the specified (blue) than in the null model (red) were retained for decomposition in the presented GLM–ASCA model (Figure S3).

**Figure S6.** Validation of GLM–ASCA model with interaction between sample type (organ‐chemotype combination) and ontogeny as fixed effect, showing (a) significance of model terms; (b) univariate model fit (pseudo‐*R* ^2^) for every model variable; and (c) variable importance (Vector Norm) for every model variable. Only responsive variables with higher pseudo‐*R* ^2^ in the specified (blue) than the null model (red) were retained for decomposition in the presented GLM–ASCA model (Figure S4).

**Figure S7.** Total ion current for SG‐associated features (TIC_SG_; x‐axes) in relation to predictions from linear mixed models (LMMs; see Fig. 5) for overall (a) and group‐specific (b) Margalef richness, and overall (c) and group‐specific (d) Pielou evenness. Black lines represent ±SE of mean. Symbol shape represents ontogeny (diamond: vegetative plants; asterisk: flowering plants); fill colour and line type represent organ‐of‐origin (light yellow triangle: adventitious roots, green dot: leaves, purple square: stems) and symbol outline colour represents leaf chemotype (yellow: saturated steroidal glycosides (SGs), blue: unsaturated SGs). Horizontal and vertical lines represent ±SE of mean for TIC_SG_ and predicted SG indices, respectively. Significant Pearson correlation coefficients are shown with R_L_ and R_S_ for leaves and stems, respectively.

**Table S1.** Estimates, confidence intervals (CI), test statistic (*χ* ^2^), and *P*‐values (*P*) of linear mixed models for Margalef's richness and Pielou's evenness for steroidal glycosides (SGs) found in extracts of different *S. dulcamara* organs. For Margalef's richness, fixed effects were modelled as the three‐way interaction between ‘leaf chemotype’, ‘organ’, and ‘ontogeny’.
**Table S2.** Analysis of deviance table (Type‐III Wald *χ* ^2^‐tests) for models presented in Figure 4 and Table S1. Test statistic (*χ* ^2^), and *P*‐values (*P*).

## Data Availability

Data (including metadata) presented here are publicly available through Zenodo (10.5281/zenodo.11080314). Code will be made available through GitHub (https://github.com/redouanadam/CH1).

## Footnotes

1 [Correction added on 21 December 2024, after first online publication: In the second sentence the word unsaturated has been changed to saturated in this version.]
