# Supplementary material for: Ontogeny and organ‐specific steroidal glycoside diversity is associated with differential expression of steroidal glycoside pathway genes in two Solanum dulcamara leaf chemotypes
Source: Plant Biol (Stuttg). 2024 Aug 16;27(5):651–68. doi: 10.1111/plb.13704 (PMC12255278; doi:10.1111/plb.13704)
Supplement: Supplementary file 9 — Table S1. Estimates, confidence intervals (CI), test statistic (χ 2), and P‐values (P) of linear mixed models for Margalef's richness and Pielou's evenness for steroidal glycosides (SGs) found in extracts of different S. dulcamara organs. For Margalef's richness, fixed effects were modelled as the three‐way interaction between ‘leaf chemotype’, ‘organ’, and ‘ontogeny’. Table S2. Analysis of deviance table (Type‐III Wald χ 2‐tests) for models presented in Figure 4 and Table S1. Test statistic (χ 2), and P‐values (P). [file PLB-27-651-s003.docx]

**Table S1**: Estimates, confidence intervals (CI), test statistic (*Χ^2^*), and P-values (*p*) of linear mixed models for Margalef’s richness and Pielou’s evenness for steroidal glycosides (SGs) found in extracts of different *S. dulcamara* organs. For Margalef’s richness, fixed effects were modelled as the three-way interaction between ‘leaf chemotype’, ‘organ’, and ‘ontogeny’. ‘Plant individual’ nested within ‘genotype’ was modeled as a random effect. For Shannon’s evenness, the three-way interaction term was not significant and therefore, it was not used as a predictor in the model.

|  | **Margalef’s richness** | | | | **Pielou’s evenness** | | | |
| --- | --- | --- | --- | --- | --- | --- | --- | --- |
| *Predictors* | *Estimates* | *CI* | *Χ^2^* | *p* | *Estimates* | *CI* | *Χ^2^* | *p* |
| (Intercept) | 2.66 | 2.58 – 2.73 | 71.74 | **<0.001** | 0.88 | 0.87 – 0.90 | 126.67 | **<0.001** |
| Chemotype [Unsaturated] | 0.14 | 0.03 – 0.24 | 2.60 | **0.011** | -0.04 | -0.06 – -0.02 | -3.81 | **<0.001** |
| Organ [Adv. Roots] | -0.27 | -0.36 – -0.18 | -5.78 | **<0.001** | 0.01 | -0.01 – 0.03 | 1.09 | 0.278 |
| Organ [Stems] | 0.01 | -0.12 – 0.13 | 0.10 | 0.921 | -0.02 | -0.04 – 0.01 | -1.19 | 0.238 |
| Ontogeny [Flowering] | 0.42 | 0.29 – 0.55 | 6.39 | **<0.001** | 0.03 | 0.00 – 0.05 | 2.24 | **0.028** |
| Chemotype [Unsaturated] × Organ [Adv. Roots] | -0.13 | -0.26 – -0.00 | -1.99 | **0.050** | 0.03 | 0.01 – 0.05 | 2.42 | **0.018** |
| Chemotype [Unsaturated] × Organ [Stems] | 0.27 | 0.09 – 0.45 | 2.93 | **0.004** | -0.06 | -0.10 – -0.03 | -3.34 | **0.001** |
| Chemotype [Unsaturated] × Ontogeny [Flowering] | -0.17 | -0.36 – 0.02 | -1.83 | 0.071 | 0.04 | 0.01 – 0.07 | 2.75 | **0.007** |
| Organ [Adv. Roots] × Ontogeny [Flowering] | 0.35 | 0.16 – 0.53 | 3.70 | **<0.001** | -0.05 | -0.07 – -0.02 | -3.33 | **0.001** |
| (Chemotype [Unsaturated] × Organ [Adv. Roots]) × Ontogeny [Flowering] | 0.24 | -0.01 – 0.49 | 1.87 | 0.064 |  |  |  |  |
| **Random Effects** | | | | | | | | |
| σ^2^ | 0.02 | | | | 0.00 | | | |
| τ_00_ | 0.01 _Genotype:Plant_ | | | | 0.00 _Genotype:Plant_ | | | |
| ICC | 0.31 | | | | 0.12 | | | |
| N | 7 _Genotype_ | | | | 7 _Genotype_ | | | |
|  | 50 _Plant_ | | | | 50 _Plant_ | | | |
| Observations | 104 | | | | 104 | | | |
| Marginal R^2^ / Conditional R^2^ | 0.814 / 0.872 | | | | 0.393 / 0.467 | | | |

**Table S2**: Analysis of Deviance Table (Type-III Wald *Χ^2^*-tests) for models presented in Figure 4 and Table S1. Test statistic (*Χ^2^*), and P-values (*p*).

|  | **Margalef’s richness** | | **Shannon’s evenness** | |
| --- | --- | --- | --- | --- |
| *Predictors* | *Χ^2^* | *p* | *Χ^2^* | *p* |
| (Intercept) | 5146.68 | **<0.001** | 16045.50 | **<0.001** |
| Chemotype | 6.74 | **0.009** | 14.52 | **<0.001** |
| Organ | 33.43 | **<0.001** | 2.91 | 0.233 |
| Ontogeny | 40.89 | **<0.001** | 5.00 | **0.025** |
| Chemotype x Organ | 12.54 | **0.002** | 22.48 | **<0.001** |
| Chemotype x Ontogeny | 3.33 | 0.068 | 7.54 | **0.006** |
| Organ x Ontogeny | 13.68 | **<0.001** | 11.08 | **0.001** |
| Chemotype x Organ x Ontogeny | 3.51 | 0.061 |  |  |
